# Covariation of Peptide Abundances Accurately Reflects Protein Concentration Differences*[Fn FN2]

**DOI:** 10.1074/mcp.O117.067728

**Published:** 2017-03-16

**Authors:** Bo Zhang, Mohammad Pirmoradian, Roman Zubarev, Lukas Käll

**Affiliations:** From the ‡Department of Medical Biochemistry and Biophysics, Karolinska Institutet, Scheeles väg 2, SE-17177 Solna, Sweden;; §Department of Laboratory Medicine, Karolinska University Hospital Huddinge, SE-14186 Huddinge, Sweden;; ¶Science for Life Laboratory, School of Biotechnology, Royal Institute of Technology-KTH, SE-17165 Solna, Sweden

## Abstract

Most implementations of mass spectrometry-based proteomics involve enzymatic digestion of proteins, expanding the analysis to multiple proteolytic peptides for each protein. Currently, there is no consensus of how to summarize peptides' abundances to protein concentrations, and such efforts are complicated by the fact that error control normally is applied to the identification process, and do not directly control errors linking peptide abundance measures to protein concentration. Peptides resulting from suboptimal digestion or being partially modified are not representative of the protein concentration. Without a mechanism to remove such unrepresentative peptides, their abundance adversely impacts the estimation of their protein's concentration. Here, we present a relative quantification approach, Diffacto, that applies factor analysis to extract the covariation of peptides' abundances. The method enables a weighted geometrical average summarization and automatic elimination of incoherent peptides. We demonstrate, based on a set of controlled label-free experiments using standard mixtures of proteins, that the covariation structure extracted by the factor analysis accurately reflects protein concentrations. In the 1% peptide-spectrum match-level FDR data set, as many as 11% of the peptides have abundance differences incoherent with the other peptides attributed to the same protein. If not controlled, such contradicting peptide abundance have a severe impact on protein quantifications. When adding the quantities of each protein's three most abundant peptides, we note as many as 14% of the proteins being estimated as having a negative correlation with their actual concentration differences between samples. Diffacto reduced the amount of such obviously incorrectly quantified proteins to 1.6%. Furthermore, by analyzing clinical data sets from two breast cancer studies, our method revealed the persistent proteomic signatures linked to three subtypes of breast cancer. We conclude that Diffacto can facilitate the interpretation and enhance the utility of most types of proteomics data.

Mass spectrometry-based proteomics is the preferred technology for quantitative and comprehensive analysis of proteins in complex biological mixtures (1). Because a typical experiment involves proteolytic digestion, the actual analytes measured by liquid chromatography-tandem mass spectrometry (LC-MS/MS)^1^ are the proteolytic peptides of the analyzed proteins. Inferring the identity of proteins that were present in the original mixture before digestion is problematic, especially when proteins are homologs. This cannot be solved by increasing the mass accuracy of measuring peptide molecules and fragments ions (2). Currently, there is no consensus concerning how such protein inference should be performed (3–5).

Further complications arise when estimating relative protein concentrations from multiple measurements of peptides. A common assumption is that the peptide abundances are proportional to their source protein's concentration (6). Thus, it is common practice to estimate a protein's concentration by the average or aggregate of its constituent peptides' abundances (7–9). Theoretically, the peptide mixture obtained from an individual protein is equimolar; however, in reality, the measured peptide abundances span several orders of magnitude. Besides, many factors can violate the assumption of proportionality. For instance, individual peptides might be subject to insufficient enzymatic cleavage or inefficient ionization; fall outside the detection range of the instrument; carry unanticipated sequence variants and modifications; share the sequence with peptides from other proteins; or might fail to be measured in some of the experiments (10). Therefore, for many proteins, the quantitative data on constituent peptides are incomplete and sometimes incoherent. To remedy this, some studies propose advanced algorithms employing powerful statistical methods (11–13), or conducting a peptide-centric analysis to avoid the inference problem (5, 14, 15).

Nonetheless, most traditional methods do not make use of the covariation of peptide abundances measured under different conditions. By putting more trust in peptides that demonstrate a stronger covariation with the other peptides from the same protein, one can make better use of the proportionality principle. Utilizing such information about covariation, other approaches have been shown to improve the validity of protein inference and signal integration (16–18), or provide a basis for selecting peptides for quantitative analysis (19, 20). However, these approaches have drawbacks in terms of dependences toward specific quantification techniques or the difficulty with handling missing values; and often incorrectly treat all peptides as independent variables when summarizing each individual LC-MS/MS experiment.

Encountered in proteomics, the problem with peptide signal integration has actually an analog in transcriptomics. Particularly, in gene expression microarrays, the biomolecules of interest are full transcripts, whereas the technology measures multiple moieties of the transcripts, *i.e.* probes (11, 21, 22). Recent technological advances in LC-MS/MS have brought proteomics to a state where its proteome coverage is comparable to that of microarrays (6, 23, 24). Although the selected sets of probes in a microarray experiment may exhibit varying affinity and genome-wide specificity (25), the veracity of the target transcripts is seldom questioned. One might then ask why proteomics, which also has multiple measurements for every target protein, requires every reporter peptide to be attributed to the source protein uniquely and be correctly identified by MS/MS, preferably in every sample. Such stringent requirements might provide a false sense of security, as it is easy to believe that correct identifications are well-suited for quantification. However, the actual relation between peptide identification and quantification may very well be reversed: as was found in our previous study (26), well-characterized chromatographic features have a better chance to be associated with correct peptide identities. In any case, the rate of false association between peptide identity and peptide quantity has not been fully investigated, and this issue is often ignored altogether. With the increasing sample sizes in proteomics studies, the impact of false quantifications may aggregate into a nonnegligible magnitude, which may affect the outcome of studies.

Fortunately, the problem with quantitatively aggregating multiple reporters into a single readout has been thoroughly investigated in microarray analysis for decades, and a set of well-characterized procedures have been developed (25, 27, 28). We argue that those hard-earned insights from microarray analysis can also be applied in proteomics to improve its quantification accuracy. In particular, we propose a differential analysis approach that we dubbed Diffacto. A popular Bayesian factor analysis algorithm (28, 29) has been implemented in this approach to handle incoherent reporter behaviors. The factor analysis extracts differential signals by utilizing the covariation over multiple experiments of abundances of a group of correlated peptides tentatively linked to a dominant proteoform.

Contrary to the popular principal component analysis, factor analysis strives to explain the covariance between observables rather than the variance within the observables, because the latter is mainly caused by random noise. In this regard, factor analysis explicitly assumes the presence of noise, and thus is more elaborate than principle component analysis. The signal (factor) represents the protein concentration change that is extracted from the correlations of measurements across multiple conditions. The signal-to-noise ratio (S/N) is then estimated for every group of peptides attributed to a single protein, to determine whether this group is informative, or too contradictory to reliably quantify. The informative groups of peptides may still contain incoherent peptides whose signals contradict those of other peptides. Such peptides are eliminated from the group before estimating the relative difference in protein concentration as a weighted geometric mean of differences in abundance of the peptides. By eliminating uninformative groups and incoherent peptide data, Diffacto reduces noise while preserving the quantitative signal largely intact, thereby allowing one to extract more useful biological information from the same proteomics data set. We demonstrate that Diffacto is a robust, sensitive and flexible method for differential proteome analysis, well suited for quantification-centered proteomics (26).

## EXPERIMENTAL PROCEDURES

### 

#### 

##### Experimental Settings for Label-free LC-MS/MS

An Orbitrap Q-Exactive Plus mass spectrometer was connected to an ultrahigh performance LC system (50-cm EASY-Spray column driven by an EASY-nLC 1000 pump), all instruments produced by Thermo Fisher Scientific (Bremen, Germany). Each sample was injected three times and analyzed in single-shot experiments with 80 min LC gradient, where the primary full-range (*m*/*z* 375 to 1400 Th) MS spectra were acquired with high resolution (140,000). Following every primary MS spectrum, one secondary MS spectrum (resolution 17,500) was acquired in a constricted *m*/*z* range (375–481, 479–601, or 599–1400 Th) for triggering data-dependent acquisition (top-10 DDA, dynamic exclusion 15 s) of tandem mass spectra (resolution 17,500). This segmented DDA approach (30) minimized the redundancy of MS/MS spectra between the three LC-MS/MS runs. To increase peptide identification efficiency by multiplexing MS/MS spectra of cofragmenting peptides (31), precursor isolation windows in the three runs were set to 2.0, 4.0 and 6.0 Th, respectively; normalized collision energy (NCE) for higher-energy collision dissociation (HCD) was set to 29 eV, 30 eV, and 31 eV, respectively. The choices of window widths and energy were based on empirical knowledge about optimal instrument settings (24), and the consideration about the density of precursors in the corresponding *m*/*z* ranges.

##### The Standard-mixture Benchmark Data Set and Label-free Data Processing

Standard digests (purchased from Promega, Madison, WI) of human cell lysates, yeast cell lysates and bovine serum albumin (BSA) were mixed at twenty different ratios (supplemental Table S1). The proportion of human peptides was reduced linearly, whereas the fraction of BSA peptides increased exponentially, and the share of yeast peptides increased nonlinearly so that all samples had equal total amounts of peptides. In each sample, 5.0 μg of peptide mixture was dissolved to a 30 μl solution, of which 6 μl were injected three times in a LC-MS/MS experiment (*i.e.* 1.0 μg peptides per injection). Raw and converted data were deposed to MassIVE (MSV000079811) and ProteomeXchange (PXD004308).

##### Peptide Identification

We identified peptides using the DeMix workflow (31), in which the MS/MS spectra were de-multiplexed by matching the isolation windows with the chromatographic feature maps generated using the full-range (survey) MS spectra by OpenMS FeatureFinderCentroided (ver. 2.0) (32). MS/MS spectra with the original and extended precursor information were searched independently in a concatenated UniProt (33) reference proteome database (6720 yeast protein sequences of release 2015_12, 91618 human protein sequences of release 2015_07, and the sequence of BSA UniProt_ID P02769) using Morpheus search engine (ver. 165) (34). Carbamidomethylation of cysteine was set as a fixed modification, and oxidation of methionine was considered as a variable modification. The target-decoy approach was applied and one missed tryptic cleavage was allowed (no proline rule). Precursor and product mass tolerances were set to 6 ppm and 18 ppm, respectively. The resulting peptide-spectral matches (PSMs) were filtered by q-value (<1%) for each individual run.

##### Peptide Quantification

Peptide-level identification and quantification were integrated through the DeMix-Q workflow (26), in which peptide chromatographic features were peak-picked from the full-range (primary) MS spectra and tentatively associated with available PSMs using OpenMS IDMapper (ver. 2.0) (32). Thereafter, the MapAlignerPoseClustering (ver. 2.0) was applied (with maximum 180 RT difference and 5 ppm precursor mass difference) to align all feature maps to the reference run (the run with the largest number of peptide-like chromatographic features), and calibrate RT to a similar scale. Subsequently, FeatureLinkerUnlabeledQT (ver. 2.0) was used to link chromatographic features across different LC-MS/MS runs and generate a consensus feature map. The consensus map provided the base for the subsequent identity propagation, where peptide identities were transferred from runs with PSM information to runs without the MS/MS information. To further increase the coverage of quantitative information, a more sensitive (extracted ion chromatography, XIC-based) signal extraction was applied by EICExtractor (ver. 2.0). Quantities from XIC were propagated to the runs where the features were not initially covered by the consensus map but precursor mass peaks at a given retention time and m/z window around the consensus feature (60 s and 5 ppm) were detected. An estimated 5% feature-level FDR was applied as a quality threshold for this process (26). If a consensus feature was linked to PSMs with different sequences, only the most common sequence was kept. Peptide abundances were reported as a sum of feature abundances from all charge-state and modifications forms of the respective sequences and normalized by the average of valid measurements of peptide abundances for each individual run.

##### Clinical Breast Cancer Proteomics Data Sets

Peptide identification and quantification results were obtained from the supplemental Materials of two clinical studies without re-processing mass spectrometry data. (1) *The CPTAC breast cancer* data set was acquired from the CPTAC study (Mertins *et al.* 2016) (35). This set was normalized in a similar approach as the original study. Peptide iTRAQ log-ratios (in relation to the internal reference) of 80 (77 samples and 3 replicate measurements) breast cancer samples (quality control passed), were normalized by kernel density estimation of two-component Gaussian mixture models, and zero-centered by subtracting the mean log-ratio of the major Gaussian distribution. Peptides quantified in no more than 30 samples were discarded. (2) *The MPIB breast cancer* data set was acquired from the original study conducted at the Max Planck Institute of Biochemistry, Germany (Tyanova *et al.* 2016) (36). Peptide ratios of 40 breast cancer samples (light, L), compared with a spike-in standard of SILAC-labeled mixture of breast cancer cell cultures (heavy, H), were log-transformed, then normalized and zero-centered by kernel density estimation of two-component Gaussian mixture models. As the original data contain H/L ratios (*i.e.* reference-to-sample ratios), we reversed the order of comparison by taking the negative values after log-transformation. Hence, the sample-to-reference comparisons were in accordance with the CPTAC data. Peptides quantified in less than 12 samples were discarded.

##### The Linear Model For Relative Protein Quantification

LC-MS/MS measured ion abundances of a protein's constituent peptide ions do not directly scale ratio to the actual amount of the original protein molecule, because of the limited efficiencies of proteolytic digestion and electrospray ionization (ESI). However, the abundances of peptides ions should be proportional to the protein concentration, if we are given fixed ionization efficiencies of the peptides. The linear dynamic range (in ESI) of peptides' responses to the difference in protein concentration, without considering the effect of charge competition in complex samples, was estimated to span over four orders of magnitude (37). Therefore, within the linear dynamic range and the limit of detection, a quantitative measurement of a proteolytic peptide should yield a readout *y* that is determined by the ionization efficiency α, the protein concentration z and the error of measurement *e*.

(Eq. 1) y=αz+e

Conventionally, peptides with highest ionization efficiency are called “best flyers”, and often used to approximate the protein concentration in the samples (38). This estimation would be reasonably accurate only when α is close to 1.0 and *e* is independent of the concentration *z* for each of (or at least most of) the peptides. But in reality, the ionization efficiencies of different peptides vary greatly, and the error of measurement depends on the peptide concentration in most deep proteomics studies.

Most often, the goal in comparative proteomics is to detect relative changes in protein concentration between biological conditions. In such cases, the ratios (instead of the actual protein concentrations) between samples are of real interests. Hence, the linear model can be formed as a comparison of two measurements, *y* and *y′*.

(Eq. 2) $$\frac{y}{y\prime }=\frac{\alpha z+e}{\alpha z\prime +e\prime }\approx \frac{z}{z\prime }$$

A log-transformation could stabilize the estimation when the error term *e* is large but the ionization factor α is relatively small:

(Eq. 3) $$\begin{array}{c} \log\left(y\right)-\log\left(y\prime \right)=\log\left(\alpha z+e\right)-\log\left(\alpha z\prime +e\prime \right)\\ =\log\left(z\right)-\log\left(z\prime \right)+\log\left(\frac{\alpha z+e}{z}\right)-\log\left(\frac{\alpha z\prime +e\prime }{z\prime }\right)\\ =\log\left(z\right)-\log\left(z\prime \right)+\log\left(\frac{yz\prime }{zy\prime }\right) \end{array}$$

The error term log(*y′z/zy′*) is assumed to be of a zero-centered Gaussian distribution that fulfills the assumption for both factor analysis and ANOVA. Hence, in this study, all peptide abundances (*y*) were rescaled by comparing to a common reference (*y′*). For the LFQ data, peptide abundances from the extra mixture were used as the reference; the CPTAC data provided an internal reference (pooled sample) for scaling; peptide abundances in the MPIB data were originally recorded in relative scale (*i.e.* H/L ratios).

##### Unsupervised Factor Analysis

A widely used Bayesian factor analysis algorithm, FARMS from Bioconductor (28), was re-implemented as a Python function. The main assumption in the factor analysis was that the peptide concentrations after digestion were proportional to the protein concentration in the undigested sample. Based on the linear model, for a given relative protein concentration *z* in log-scale, zero-mean normalized observations of log-ratios of abundances ***x*** = log(**y**) − log(**y′**), hereafter referred to as abundances, from constituent peptides could be described as ***x*** = λ*z* + **ε**. According to the definition in FARMS, the vector λ describes the contributions of individual peptide signals, and the vector **ε** stands for the noise caused by errors of measurements. By assuming that *z* and **ε** are independent, factor analysis can be employed, with *z* as a factor and λ as loadings. Furthermore, the observation ***x*** should follow a Gaussian distribution: ***x***∼
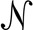
*(0,* **λλ***^T^* + **Ψ***)*. Here, **λλ***^T^* stands for the peptides' signal covariance matrix, whereas i**Ψ** represents a diagonal noise covariance matrix. Our goal is to find the nonnegative *maximum a posteriori* estimation of loadings **λ** that best describes the covariation of abundances **x**, and to estimate for every protein the S/N by comparing the signal **λ** and noise **ε** for calling the set of peptides either informative or noninformative (29). Algorithmic details can be found in the references Hochreiter *et al.* (28) and Talloen *et al.* (29).

The factor loadings **λ** can be considered as peptides' responsiveness to protein concentration changes and used as weighting factors as well as quality control indicators in signal integration. Peptides weighted lower than half of the maximum weight were considered falsely identified or unreliably quantified, and thus were disqualified from signal integration. This quality threshold enabled auto-exclusion of unreliable peptides, which then increased the overall robustness of protein quantification.

##### Relative Protein Quantification

Given the labels of samples, the relative abundance of a protein is calculated as a weighted geometric mean (*i.e.* a weighted arithmetic mean in log-scale) of abundances of constituent peptides (w > 0.5) for the entire group of experiments (samples).

(Eq. 4) $$\log\left(z/z\prime \right)=\frac{\sum_{\text{i}=1}^{n}w_{i}\left(\log\left(y_{i}\right)-\log\left(y_{i}\prime \right)\right)}{\sum_{\text{i}=1}^{n}w_{i}}$$

The weight of each peptide (*w_i_*) was given by the factor loading from the previous unsupervised analysis. This method also addressed the missing value problem by simply omitting these peptides' contributions to the geometric mean. However, in some cases missing values indicated abundances below the detection limit, and could carry information when frequently observed in a specific sample group. Hence, for the LFQ data, missing value imputation was applied when a large fraction (>70%) of peptide measurements from one sample was missing. In that case, missing values in that sample were filled with half of the lowest registered abundance for the set of peptides.

##### Analysis of Variances (ANOVA)

was built on the same assumption as in the unsupervised factor analysis: linear signals plus Gaussian noise. Given a protein in *I* conditions with *J* experiments and *K* constituent peptides qualified by the factor analysis (*w* > 0.5), we denoted the per-group estimated relative abundances *x̂_i_* and the average abundance *x̄* for peptides (all in log-scale). The total sum of squares (TSS), the residual sum of squares (RSS) and the explained sum of squares (ESS) can be expressed as

(Eq. 5) $$\begin{array}{c} TSS=\sum_{i=1}^{I}\sum_{j=1}^{J}\sum_{k}^{K}\left(x_{ijk}-\bar{x}\right)\\ RSS=\sum_{i=1}^{I}\sum_{j=1}^{J}\sum_{k=1}^{K}\left(x_{ijk}-{\hat{x}}_{i}\right)\\ ESS=TSS-RSS \end{array}$$

ESS has *I* − 1 degrees of freedom; RSS has, in principle, *IJK* − *I* degrees of freedom. However, missing values should be excluded from the calculations, which accordingly reduces *m* (number of missing values) degrees of freedom in RSS. Hence, the *F*-statistics can be formulated as

(Eq. 6) $$F=\frac{\frac{ESS}{I-1}}{\frac{RSS}{IJK-I-m}}$$

This expression should, in principle, follow an *F*-distribution with (*I* − 1, *IJK* − *I* − *m*) degrees of freedom. A *p* value could then be calculated for testing the null hypothesis (H_0_: all sample groups having the same mean *peptide* abundance). Unfortunately, although such a peptide-level statistical approach is extremely sensitive, the distribution of quantification errors might be non-Gaussian because of the covariation of peptides, which could violate the assumption of ANOVA. A typical example is having batch effects caused by outlier samples. In that case, rejecting H_0_ at the peptide-level might not have the same meaning at the protein-level (supplemental Discussion). In order to control the risk of inflating significance at the protein level, without reintroducing the sample-wise protein quantification, two types of tests were applied to estimate the significances of differentially expressed proteins: the FDR_MC_ approach (39) and the PECA approach (15).

*Sequential Monte Carlo multiple testing (FDR_MC_)* (39) was applied when the sample size is large enough to generate hundreds of thousands of permutation sequences. A batch (100 times) of randomized shuffling of sample labels was generated for each iteration of Monte Carlo (MC) simulation. The ESS (or the F-statistic) for each informative protein (S/N > −20 dB) was calculated based on the grouping with the randomized sample labels. Denoting *T* as the number of simulations that yield equal or greater ESS than that obtained with the true labels and the estimated per-group abundances *x̂_i_* from the weighted averaging; and *N* as the total number of MC simulations for the given protein, MC *p* values could be calculated as follows:

(Eq. 7) $$P_{mc}=\frac{T+1}{N+1}$$

After each batch of MC simulations, a set of q-values was estimated based on ascending ordered *P_mc_* values and a conservative estimation of π_0_ (proportion of true null hypotheses) (40).

(Eq. 8) $${\hat{\pi }}_{0}=\min\left(1,\frac{2}{m}\sum_{i=1}^{m}P_{mc}^{\left(i\right)}\right)$$

(Eq. 9) $$q^{\left(i\right)}=\underset{\text{i}\leq \text{j}\leq \text{m}}{\min}\left(m\cdot {\hat{\pi }}_{0}\cdot \frac{P_{mc}^{\left(i\right)}}{j}\right)$$

The MC simulation stopped when *T* ≥ 200 for a given protein, or q-values of all remaining proteins are lower than the 0.05 FDR threshold.

*Adaptation of the PECA approach* (15), on the other hand, tests the null hypothesis individually for each of the constituent peptides of a protein. Based on the notation, set *x̂_i_* = *x̄_ik_*, the F-statistic for the *k*-th peptide equals:

(Eq. 10) $$F_{k}=\frac{\frac{\overset{I}{\sum_{i=1}}\overset{J}{\sum_{j}}\left(x_{ijk}-{\bar{x}}_{ik}\right)}{I-1}}{\frac{\overset{I}{\sum_{i=1}}\overset{J}{\sum_{j=1}}\left(x_{ijk}-{\bar{x}}_{k}\right)-\overset{I}{\sum_{i=1}}\overset{J}{\sum_{j=1}}\left(x_{ijk}-{\bar{x}}_{ik}\right)}{IJ-1-m_{k}}}$$

The peptide-level *p* value can be calculated by the cumulative distribution function for the F-distribution (*f*_cdf_).

(Eq. 11) $$P_{k}=1-f_{cdf}\left(F_{k},I-1,IJ-1-m_{k}\right)$$

Under the null hypothesis, the median of uniformed distributed *p* values should form the order statistics with a beta distribution (α = β = (*k* + *1*)/2). Hence, the protein-level significance was determined by the median of peptide-level *p* values (P_k_*) and the cumulative distribution function of the beta distribution (*B*_cdf_).

(Eq. 12) $$P=B_{cdf}\left(P_{k}^{*},\frac{K+1}{2},\frac{K+1}{2}\right)$$

Similarly, q-values for the PECA approach were estimated using the same formula as in the FDR_MC_ approach. However, we found the peptide-level statistics might not be well-calibrated that still tend to overestimate the significance at the protein level, thus must be applied with cautions (supplemental Discussions).

##### Comparison to Traditional Per-Sample Quantification Methods

(*a*) The Top-3 approach summarized each protein by taking the arithmetic mean abundance of its three most abundant peptides (or all its peptides when having less than three peptides). This approach is often applied as the “gold-standard” with the assumption that “best flyer” peptides give better MS responses (38). (*b*) The Median approach used the median values of nonmissing peptide measurements to make per-experiment estimations of protein abundances, which is commonly applied in studies with paired samples and using isotopic labeling quantification. (*c*) The PQPQ approach was adapted from the simplified version (20) written by Zhu *et al.* Peptides abundances were log-transformed then filled with zeros for missing values. For each protein, peptides were clustered based on the pattern of correlation across samples, using the hierarchy linkage function with default settings for distance calculating method (“complete”), metric ('correlation') and threshold (0.4). The largest cluster from PQPQ was chosen as the representative of the protein, and combined by taking the average abundance. (*d*) The MaxLFQ algorithm was implemented in MaxQuant (12, 41), which took the Thermo .raw files as input, processed the data with the Andromeda search engine, and propagated peptide identifications via the option of “match between runs”; 1% FDR of PSM and 5% FDR of proteins were allowed. Protein abundances for each sample were given by averaging the reported nonzero abundances from the three LC-MS/MS experiments. Proteins quantified in less than five samples were discarded.

##### False Quantification Rate in LFQ

To test the impact of false peptide quantifications aggregated on the protein level, the correlation between the known concentration and the protein level quantification was measured. For each protein, the ranks of 190 pairwise abundance ratios (formed from the 20 mixtures) were compared via Spearman's rank correlation between the reference concentrations and the protein quantification results. Pair ratios involving missing values were excluded. Proteins with negative correlation (below the threshold *r* = 0) were considered as false quantifications.

##### Analysis of ABRF-iPRG-2015 Data Set Using Peptide De Novo *Sequencing*

Raw data of the 12 LC-MS/MS experiments from iPRG-2015 study were downloaded from the FTP server (ftp://iprg_study:ABRF329@ftp.peptideatlas.org) and processed as described in reference (26). Instead of performing traditional MS/MS database search, we used DeNovoGUI (ver. 1.14.5) (42) that contains the *de novo* sequencing software Novor (43) to generate full-length peptide sequences directly from the MS/MS spectra, with 10 ppm precursor mass tolerance and 15 ppm fragment mass tolerance. Carbamidomethylation of cysteine was set as a fixed modification, and oxidation of methionine was considered as a variable modification. The universal SwissProt database (release 2014_07 containing 546,000 protein sequences) (33) was searched for all the *de novo* peptides using protein BLAST (ver. 2.2.28, parameter: -task blastp-short). The top 10 BLAST hits were filtered by sequence coverage and identity, using arbitrary qualification criteria, where at least 7 identical residues covered at least 80% of the *de novo* peptide sequence, and the overall identity was higher than 80%. After quality control, *de novo* peptides were considered as MS/MS identifications, and were assigned to the chromatographic feature maps by the DeMix-Q workflow. Multiple peptides were allowed to be associated with the same features, because of the uncertainty of *de novo* sequences. In addition, each *de novo* sequence was allowed to have multiple BLAST matches to the proteins that are homologs from different organisms. Therefore, the species identification codes from the SwissProt entry names of the matched proteins were removed. For instance, OVAL_CHICK and OVAL_MELGA were considered as the same source protein with the identification code of OVAL. As a result, the quantification table contained peptide sequences that mapped to conceptual source proteins and were quantified in at least 4 of the 12 experiments. Finally, Diffacto analysis was performed based on the resulting peptide quantifications, with below-threshold FDR calculated based on 500,000 random Monte Carlo permutations.

##### Code Availability

Source code (Python 3.x) and examples for Diffacto are freely available at https://github.com/statisticalbiotechnology/diffacto under Apache 2.0 license. Package dependences: scipy, numpy, pandas (http://pandas.pydata.org), networkx, Pyteomics (44), and Scikit-learn (45).

## RESULTS

### 

#### 

##### Software Implementation of Diffacto

The aim of Diffacto is to detect proteins that have different concentrations between samples and to quantify such differences. By comparing each sample to a common reference, the input lists of peptide abundances (*x*) were firstly transformed to a relative scale (*i.e.* log-ratios). The transformation of abundance scale balanced the contributions of peptides for each protein, despite the vastly different ion-intensities observed in LC-MS/MS experiments. Therefore, based on the proportionality principle, every observed peptide abundance should be a combination of two parts: the signal responding to the relative change of protein concentration (*z*), plus the noise (**ε**) mainly caused by measurement errors. Given more than one peptide observations for a protein, each individual peptide could be weighted by a parameter (**λ**), depending on the covariation between each other peptides and the estimated noise **ε**. Thus, the linear model was described as: ***x*** = **λ***z* + **ε**. Assuming statistical independence between *z* and **ε**, a factor analysis could be employed with *z* as a factor and **λ** as loadings, the latter provided means to assess the reliability of each peptide and maximize the extraction of the signals of protein concentration changes.

We re-implemented a Bayesian factor analysis method, FARMS (28), as a Python function. The method was originally intended for the analysis of gene expression microarrays, but we redressed the method to determine common components of abundance variation from a set of peptides that are tentatively linked to one proteoform. The factor analysis dissected the covariance of measured peptide abundances **x,** Cov(**x,x**) into an assumed response to the protein concentration (*i.e.* the signal covariance matrix **λλ***^T^*) and the errors of measurements (*i.e.* the diagonal noise covariance matrix **Ψ**), assuming a Gaussian distribution: ***x*** ∼ 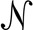
(0, **λλ**^T^ + **Ψ**). This approach rendered a *maximum a posteriori* estimation of nonnegative factor loadings λ to best describe the covariance of peptide signals; at the meantime, a S/N was also given for every set of peptides by comparing the signal to the remaining noise. We chose a modest S/N threshold of −20 dB (*i.e.* 1%) to categorize the sets of peptides into two groups: informative and noninformative (originally termed I/NI-calls) (29). Noninformative sets were excluded from the analysis of differential proteins.

We investigated the factor loadings **λ** for the informative sets of peptides and observed a bimodal distribution between 0 and 1. Thus, we applied an arbitrary selected loading threshold of 0.5 for screening individual peptides and removed peptides that are incoherent to the estimated covariance structure. The process was unsupervised and hence did not require information about sample labels or study design (*e.g.* pairwise or multi-group comparisons), a property that makes it suitable for large-scale studies with complex designs. Thereafter, using **λ** as weights, we calculated the relative protein abundances for each sample group (instead of each individual experiment) by the weighted geometric means of the relative peptide abundances.

##### Accurate Protein Quantification on Controlled Experiments

To comprehensively investigate the extent of deviating behaviors of peptides in response to protein concentration changes, we performed a set of single-dimensional label-free LC-MS/MS experiments with 20 mixtures of human, yeast and BSA standard digests combined in different proportions (supplemental Table S1). We quantified in total 38,794 peptides (supplemental Table S5) that were attributed to 4804 proteins (excluding 2318 proteins identified by single peptides) (supplemental Table S2). Applying peptide identity propagation in DeMix-Q (26) yielded an overall quantification rate of 87.9% (*i.e.* 12.1% missing values). The median CV of the peptide quantification in three replicate experiments was 12.4%. As a comparison, we also processed the data set using MaxQuant (MaxLFQ), which yielded 38,738 peptides and 3650 proteins that passed the threshold of sample coverage (were quantified in at least 15 runs for peptides or 5 samples for proteins). The MaxQuant-derived peptide quantification table contained 36.6% of missing values, which was more than three times as frequent as in the DeMix-Q output.

We assessed the linear range in the experimental conditions based on the abundances of peptides derived from BSA that spanned four orders of magnitude in the 20 mixtures (supplemental Table S1). We observed no obvious upper limit of quantification (LOQ) even for the highest amount of BSA spike-in (supplemental Fig. S1, Supplemental Note S1). The linearity observed from most of the BSA-derived peptides suggested that the sample overload was not an issue. However, for half of the samples with low BSA concentration (relative abundance less than 0.5% of the total amount), we observed a lower LOQ (supplemental Fig. S1), which determined that the linear range of the measurements covered at maximum 30 times the difference in the protein concentrations.

**Fig. 1.**
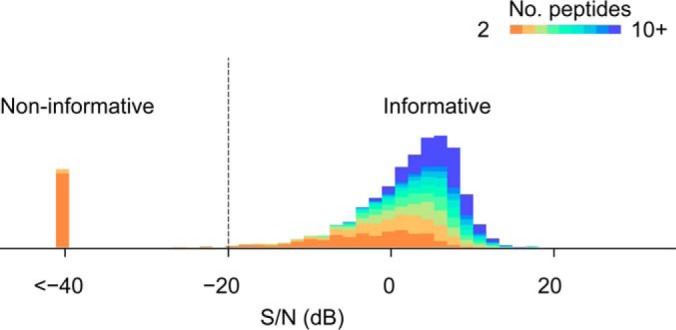
**The distribution of signal-to-noise ratios.** Estimated by the Bayesian factor analysis, the higher S/N values reflect the stronger covariations of abundances for the sets of quantified peptides. Proteins (as sets of peptides) with contradicting peptide responses (mostly with only two or three constituent peptides) were deemed noninformative (S/N < −20 dB).

For each protein, we investigated the covariation structure of its constituent peptides' abundances and categorized 91% (4365 out of 4804) of the identified proteins containing informative sets of peptides (Fig. 1). Reassuringly, the S/Ns of the summarized proteins showed a strong dependence on the number of constituent peptides, implying that improved protein coverage increases the certainty of quantification in the protein-centric aggregation. Indeed, among the 439 noninformative proteins, 346 (79%) had only two peptides whereas 70 proteins (16%) had three peptides (supplemental Table S2).

**Fig. 2.**
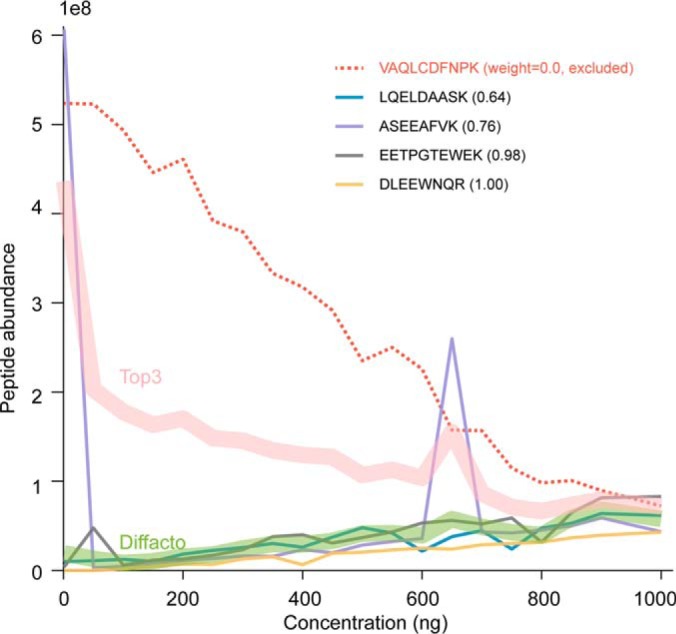
**Protein summarization based on peptide abundances (an example).** Five peptides matched to the protein CLCB_HUMAN (UniPort ID) showed varying ionization efficiencies, but the majority of the peptides responded to the actual protein concentration, which is assumed to be proportional to the fraction of human protein contents in the analyzed mixture. Concentration unit: ng per 6 μl. The peptide with the overall highest abundance (red dashed line) showed anticorrelation with other peptides. The deviating behavior could be a result of errors in the peptide identification process or the ion-chromatography extraction. Diffacto assigned a weight of zero to this particular peptide and excluded it from the signal integration. The weighted geometric mean that given by Diffacto (green band) also appeared to be more stable compared with the Top3 averaging (red band), in the presence of outliers (two spikes of the purple line).

**Fig. 3.**
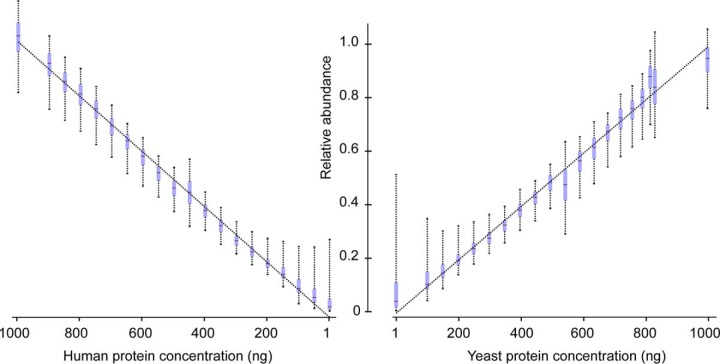
**Protein LFQ by weighted geometric means of peptide abundances.** The inter-quantile ranges (boxes) and 90% percentile range (whiskers) of relative quantifications of 2800 informative human proteins (left panel) and 1564 yeast proteins (right panel) in 20 mixtures. Concentration unit: ng per 6 μl. Relative quantification using weighted geometric mean showed tight linear relation to the actual protein concentration in the mixtures. Human proteins appeared to have smaller quantification errors compared with those of the yeast proteins, because of an on average larger number of constituent peptides per protein. Results of three other summarizing approaches are presented in supplemental Fig. S3.

In the informative sets of peptides, the covariation structures extracted by the factor analysis served as the quality control, which disqualified 11% of the identified peptides for the quantitative summarization because of their incoherent signals (Fig. 2). The quantification schema in Diffacto, *i.e.* weighted geometric means, addressed the missing value problem in label-free quantification (LFQ) by only integrating valid measurements. Unlike arithmetic means, geometric means are less affected by outlier values and are, therefore, more robust against quantification errors. As a result, we obtained linear correlations between the estimated abundances of the informative proteins in 20 mixtures and the known actual concentrations of human or yeast cell lysates (Fig. 3 and supplemental Table S2). However, for samples where a large proportion of the peptides' concentration fell below the level of detection, ignoring the hidden signals from missing values might enlarge the proportion of quantification errors (supplemental Fig. S3). For such cases, it remained necessary to impute the missing values. This was done by arbitrarily assigning half of the lowest abundance observed to the missing measurements in a given group of samples if the fraction of missing values exceeded 70%, a fraction set to be more than five times larger than the overall missing value rate for that group of samples. When using MaxLFQ derived peptide abundances, the threshold for this imputation was increased to 95%, to better accommodate the large fraction of missing values in the MaxQuant-derived output. Even under these conditions, Diffacto inferred 3955 informative proteins, and again recovered the linearity between the summarized abundances and the actual concentration, despite the more than tripled rate of missing values.

**Fig. 4.**
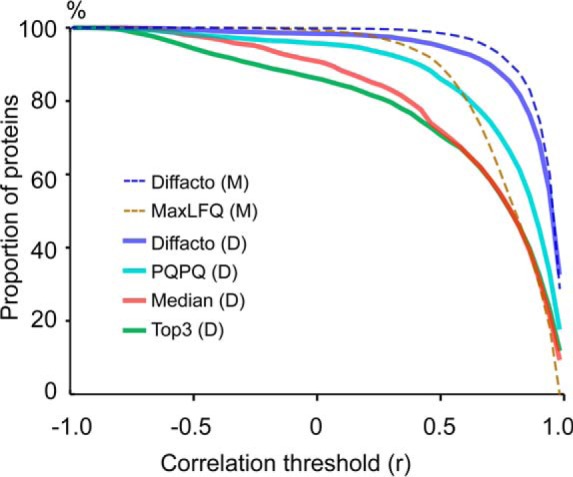
**Evaluation of the precision of protein quantification results.** Dashed lines: quantification based on MaxQuant (M) peptide abundances. Solid lines: quantification based on DeMix-Q (D) peptide abundances. Abundances of informative proteins summarized by different techniques were correlated to the actual protein concentrations. The proportions of quantified proteins (*y* axis) at the correlation threshold (*r* = 0) were used to estimate false quantification rates: 14.3%, 9.6%, 4.3%, 1.6%, 0.68%, and 0.13%, respectively in Top3 (D), Median (D), PQPQ (D), Diffacto (D), MaxLFQ (M) and Diffacto (M) results. Both Diffacto and PQPQ reduced false quantifications by the elimination of contradicting peptides. However, the weighted geometric means summarized by Diffacto provided overall higher precision than other methods. Because of the presence of contradicting peptides, Top3 appeared to be the most vulnerable approach, which performed worse than summarizing protein concentrations by their peptides with median abundances.

Because the human proteome and the yeast proteome were two independent components in the 20 mixtures, the two background proteomes provided a base for us to investigate the false quantification rate (FQR). We defined FQR as the fraction of protein quantifications correlating negatively with the actual concentrations (Methods and supplemental Note S2). The result turned out to be devastating for so-called *one-hit wonders*, *i.e.* proteins identified by only one peptide. For such proteins, the FQR was estimated at the level of 29%, not much better than the random noise that would give on average a 50% FQR. For proteins quantified by two peptides, the FQR was 19% but dropped to below 6% after the S/N filtering (*i.e.* excluding noninformative proteins). For the “gold standard” proteins with three peptides, we found the FQR to be 6% before S/N filtering and 3% after filtering. We compared the overall performance of Diffacto against other quantification approaches (Fig. 4): MaxLFQ; averaging of Top-3 most abundant (or so-called best flyer) peptides; Median of all quantified peptides; and PQPQ (19), a method that utilizes peptide correlation for clustering and quality thresholding.

MaxLFQ quantifies proteins based on linear regressions of their peptides' pairwise log-ratios, a metric that to some extent is robust in respect to quantification errors. As a result, for the 3650 proteins MaxLFQ showed an impressive overall FQR of 0.68%. However, using the same set of peptide abundances, Diffacto not only summarized 300 more proteins, but also achieved five times less FQR (0.13%), fewer missing values, and better precision (Fig. 4). On the other hand, with DeMix-Q peptide abundances, PQPQ clustered peptides into subgroups by an arbitrary threshold of linear correlation (*i.e.* a threshold not determined by the estimation error of measurement errors), which reduced the FQR compared with Top-3 and Median, especially when having a large number of correlating peptides. However, an acceptable FQR < 5% was achieved only for proteins with more than six peptides, which encompassed only one-third of the quantified proteome. In sharp contrast (supplemental Fig. S2*b* and S2*c*), Diffacto removed the major source of false quantifications (*i.e.* proteins having low coverage) by the S/N filtering. The quantified 4361 informative proteins showed an overall FQR of 1.6%. This result confirmed the vital importance of measuring multiple peptide abundances per protein.

##### Proteomic Portraits of Three Subtypes of Breast Cancer

To demonstrate the performance of Diffacto in large-scale comparative proteomics, we re-analyzed two benchmark data sets from clinical breast cancer studies, one conducted by the Clinical Proteomic Tumor Analysis Consortium (CPTAC, Mertins *et al.)* (35) and another one by the Max Planck Institute of Biochemistry (MPIB, Tyanova *et al.)* (36). The CPTAC study could be seen as a near-ideal case of comparative proteomics, which has an internal reference (a pooled sample), reasonably large sample size (77 clinical samples) and excellent sequence coverage (24 peptides per protein, on average). The MPIB study, on the other hand, represented a less ideal case in proteomics, where 40 tissue samples were compared against a standard mixture of stable-isotope labeled breast cancer cell lines using the super-SILAC approach. For consistency, we followed the same approach as in Tyanova *et al.* to categorize the samples into three groups: estrogen or progesterone receptor positive (ERPR+), epidermal growth factor receptor positive (HER2+) and ER/PR/HER2 triple-negative (TN). We also grouped the quantified peptides by source genes instead of protein sequences.

**Fig. 5.**
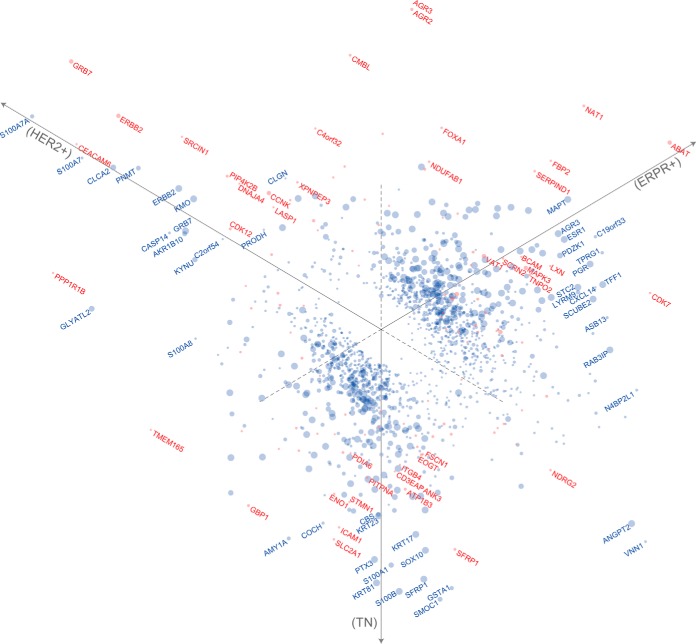
**Three-way comparisons of breast cancer subtypes from CPTAC and MPIB proteomics data.** Diffacto summarized differential proteins from CPTAC (blue) and MPIB (red) data sets. The differentially expressed proteins were more comprehensive and subtype-specific in the former data set, because of its larger the sample size and higher sequence coverage. Particularly, for the triple-negative subtype, which has been characterized by the high abundances of basal-like cytokeratins (56), SMOC1, S100B, GSTA1, SFRP1, S100A1, PTX3, SOX10, ANGPT2, COCH, as well as many other known markers such as SYNM1 (57), MFI2 (58), NDRG2 (59), CRYAB (60), and PLA2G4A (61) were found on top of the list of differential regulated proteins in the CPTAC data (supplemental Table S3). Markers are located on the scatter plot based on the fold changes relative to the three subtypes (axes) in log-scale. Marker radius is proportional to the *q* value (FDR_MC_) in negative logarithm scale.

We classified in total 7879 (92%) proteins in the CPTAC data as informative with the S/N cutoff at −20 dB, and estimated that 1470 protein concentrations (19% of the informative proteome) were significantly different (FDR<0.05) between the cancer subtypes (Fig. 5 and supplemental Table S3). This number was marginally (2%) smaller than the 1506 proteins estimated by ANOVA using median ratios, indicating that the FDR control by Monte Carlo random permutations was conservative. In the list, we found 25 proteins of the PAM50 marker genes (46), which validated their roles as protein markers. This can be compared with the 3889 (67%) informative proteins we found in the MPIB data (supplemental Table S4). Among these, only 115 proteins (3% of the informative proteome) had significantly different concentrations (q < 0.05, Fig. 5 and supplemental Table S4), despite the fundamental differences between the subtypes of cancer as observed in the CPTAC data. Nevertheless, at the same level of 5% FDR, Diffacto detected 85% more significantly differential proteins than the 62 proteins originally reported by Tyanova *et al.;* which was also more than twice as many as the 47 proteins detected by the conventional approach of applying an ANOVA to the median-summarized protein concentration ratios. Interestingly, we found that Diffacto reported a very different set of proteins than that in the original report: only 24 of the 62 original proteins were recalled as differentially expressed. We investigated this discrepancy and found among the proteins in the Tyanova study that were not reported by Diffacto, one protein that was rejected because of too many missing values, 6 proteins (including one of the markers selected by Tyanova *et al.*) were deemed noninformative, and 31 proteins were estimated not significantly different as estimated by the potentially stricter error control by Diffacto.

**Fig. 6.**
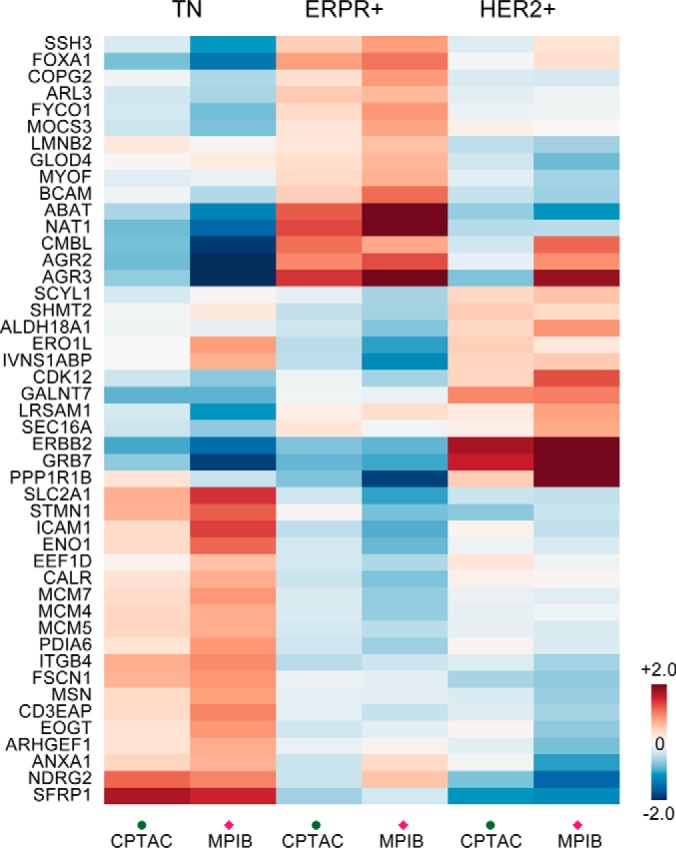
**Relative abundances of 46 differential proteins common for CPTAC and MPIB data.** Protein fold-changes estimated by Diffacto (weighted geometric means) showed good agreements not only in the directions of regulation, but also in the magnitudes of changes between (supplemental Fig. S5). Such protein expression patterns clearly clustered into three groups that represent the most persistent proteomic signatures of the three subtypes of breast cancer.

To demonstrate the Diffacto's improved quantification, we investigated the consistency of the summarized protein abundances between the CPTAC and MPIB sets. By comparing the lists of differential proteins reported by Diffacto, we found 46 proteins in common (Fig. 6), and among these, 22 were exclusively detected by Diffacto. A strong linear correlation of protein ratios (0.88) was observed between the CPTAC and MPIB data sets (supplemental Fig. S5). In contrast, for the 12 proteins that were exclusive to the original MPIB report, the correlation of median protein ratios was only 0.7 between the data sets. Importantly, the Diffacto-exclusive proteins were biologically relevant. We found among them three known markers, NAT1 (46), PPP1R1B (35) and ITGB4 (47), respectively associated with the ERPR+, HER2+ and TN subtypes; and the rest clearly characterized the TN subtype by the specific upregulations of CD3EAP, FSCN1, ICAM1, MCM4, MCM7, PDIA6, and SLC2A1 (also known as GLUT1) (Fig. 6, Supplemental Note S3).

##### Analysis of iPRG-2015 Data Using Peptide De Novo Sequencing

To demonstrate that reliable protein quantification can be obtained even with a spectacularly suboptimal identification procedure, we took the data from the iPRG-2015 study (48) for illustration. In the iPRG study, six marker proteins were spiked with different concentrations into four samples with 200 ng yeast proteins, and were subsequently measured by triplicate LC-MS/MS experiments. We made no assumptions regarding either the spiked proteins or the background proteome. Instead, we used *de novo* peptide sequencing followed by protein BLAST against the universal SwissProt database. This protocol associated each of the 15,927 chromatographic features with multiple *de novo* sequences, and grouped the latter into 1852 abstract source proteins (supplemental Table S6). Even using such an uninformed identification process, Diffacto detected and properly quantified all six spike-in proteins (Table I). Only two pairs of low concentration differences (65:55 fmol and 11:10 fmol) were not correctly quantified in the relative scale, but comparisons of the theoretical protein ratios and quantification results showed a high degree of linear correlation (supplemental Table S6). The distinctive signal-to-noise ratios of the six marker proteins confirmed the usefulness of the quantification approach based on underlying covariations of the peptide signals.

**Table I:** The 10 most differential proteins in the iPRG-2015 data. For protein identification, we used peptide de novo sequencing followed by protein BLAST search against the full SwissProt database. Despite such a relatively uninformed identification procedure, Diffacto managed to filter out wrongly identified sequences and obtained representative protein concentration estimations

Protein	No. peps	w>0.5	S/N (dB)	^a^PECA	FDR_MC_	^b^S1	S2	S3	S4	^c^REF1	REF2	REF3	REF4
**OVAL**	2	2	**18.14**	**0**	**0.03**	64.46	57.38	10.54	2.62	65	55	15	2
**BGAL**	33	17	**14.37**	**0**	**0.03**	0.44	73.96	54.84	7.76	2	65	55	15
**PYGM**	32	15	**12.67**	**0**	**0.03**	12.68	0.21	64.04	60.07	15	2	65	55
**CAH2**	15	8	**6.23**	**0**	**0.03**	13.80	495.87	11.39	0.53	10	500	11	0.6
**MYG**	4	3	**24.58**	28.8	**0.03**	71.54	8.47	0.002	56.98	55	15	2	65
PPID	8	6	-3.6	158.06	**0.03**	1.87	1.75	1.65	0.19	-	-	-	-
**ALBU**	35	19	**12.92**	**0**	0.13	4.82	0.02	4.64	512.12	11	0.6	10	500
TRM6	2	2	-8.49	134.66	0.23	1.10	1.19	0.76	1.02	-	-	-	-
ATG27	3	2	-16.04	184.92	0.23	0.99	0.99	1.18	0.87	-	-	-	-
SKI2	5	2	-2.51	10.03	0.26	1.18	1.17	0.97	0.75	-	-	-	-

^a^ family-wise error rate (Bonferroni correction of PECA *p* values).

^b^ Diffacto summarized relative protein abundances, rescaled by the average reference spike-in abundances for marker proteins, or by the median peptide abundances for background proteins.

^c^ Reference amounts (fmol) of proteins spiked in the samples, Proteins not deliberatively spiked in the samples are indicated by a null reference concentration “-”.

## DISCUSSION

High-resolution mass spectrometry has transformed proteomics by providing a greater number of identifications and more reliable quantitative measurements of proteolytic peptides, compared with earlier generations of equipment. Currently, it might seem like protein quantification being limited by the identification process of mass spectrometry data, and consequently that the best way to improve quantitative accuracy is to improve the identification process. However, this might not be the best way forward as we increase the sample sizes in LC-MS/MS experiments. Expansions in number of samples do not necessarily benefit the traditional identification process (49); but surely accumulate more quantitative information. Furthermore, identification-based quantification approaches, such as MS/MS spectral counting (SpC), have a certain limit to their accuracy because of the stochastic nature of DDA and the low average number of peptide counts. Although we would not argue against the usefulness of such methods in general, we do not recommend using Diffacto with SpC data. The application of the segmented DDA strategy in the current study rendered the traditional identification-based approaches impractical, unlike the XIC-based approaches.

As we have demonstrated in this study, the peptide abundances provide means to improve identification. The covariations of peptides' abundances captured by the factor analysis method implemented in Diffacto provided not only a quality control but also a weighted summarizing schema. Other methods for peptide weighting (18, 50) that use different aspects of peptide properties might also be applied together in this schema for improving protein quantification accuracy. This schema comes with a feature of robustness that makes the grouping of peptides more flexible, and thus reduces the burden of protein inference. An interesting extension would be to adapt Diffacto to summarize protein concentration changes based on arbitrary rules that assume covariations of peptides, such as source genes (as we applied in the breast cancer analysis), protein complexes, organelles, interactions, regulations, and pathways. We made a bold attempt, in light of our previous study (51), to test the feasibility of using *de novo* peptide sequencing and sequence homology search to analyze the iPRG-2015 spike-in type of data set. Although the data set is insufficiently large to draw a comprehensive conclusion, we obtained both high specificity and quantitative accuracy for all the spiked marker proteins (Table I and supplemental Table S6). In the current approach, the statistical significance of the protein abundance differences is derived from ANOVA, which might likely be determined by the sample with the most distinctive quantities. In this context, the statistical significance of pairwise comparisons was not given. Regardless of such limitation, this result provides an alternative way of data analysis for situations in proteomics and proteogenomics where the “reference genome/proteome” is absent.

It is not that uncommon to report lists of differential proteins in proteomics studies controlled by *p* values from pairwise t-tests. Uncorrected *p* values may lead to many false positive results in a proteome-wide analysis (52) (supplemental Discussions). Instead, FDR or *q* values have become the default in reporting identification results by MS/MS (4, 49, 53). Many studies in proteomics are limited in sample size, and hence lack statistical power to overcome the burden of multiple testing corrections (54), which frequently results in studies reporting *p* values rather than FDRs. We took a clinical data set of bladder cancer (55) as an example: many signature proteins of the muscle-invasive bladder cancer could be consistently detected by Diffacto from both LFQ and iTRAQ data (supplemental Note S4 and supplemental Fig. S6). However, because of the relatively small sample size (4 + 4), we could not calculate robust FDR_MC_ metrics. This raises a question of how many experiments are needed in order to perform a reasonable Diffacto analysis with a proper FDR control. In theory, three experiments are the minimum requirement in order to measure covariation; six samples (3 + 3) are needed for t-tests; and 10 samples (5 + 5) are the minimum for a reasonable MC random permutation test. Although the increasing sample sizes in proteomics, we will soon approach the point when *p* values should be replaced by FDRs in reporting quantification results. Perhaps it is time to recognize that we should spend more time thinking about how to correctly quantify the proteomes instead of continuing emphasizing the false identifications of peptides and proteins. Advanced protein quantification methods, such as the one suggested in this study, could well address the issue with both FDR and FQR by better utilizing the wealth of multi-dimensional information in shotgun proteomics.

## DATA AVAILABILITY

Raw and converted data are deposed to MassIVE (MSV000079811) and ProteomeXchange (PXD004308). Source code of Diffacto is freely available at https://github.com/statisticalbiotechnology/diffacto under Apache 2.0 license.

## Supplementary Material

Supplemental Data

## Footnotes

^1^ The abbreviations used are:

LC-MS/MS: liquid chromatography coupled to tandem mass spectrometry
ANOVA: analysis of variance
CV: coefficient of variation
CPTAC: Clinical Proteomic Tumor Analysis Consortium
DDA: data-dependent acquisition
ERPR: estrogen or progesterone receptor
FARMS: Factor Analysis for Robust Microarray Summarization
FDR: false discovery rate
FQR: false quantification rate
HER2: human epidermal growth factor receptor 2
iTRAQ: isobaric tag for relative and absolute quantitation
LFQ: label-free quantification
LOQ: limit of quantification
MC: Monte Carlo method
FDR_MC_: false discovery rate based on sequential Monte Carlo simulation
MPIB: Max Planck Institute of Biochemistry
PECA: Probe-level Expression Change Averaging
PQPQ: protein quantification by peptide quality control
PSM: peptide spectrum match
RT: retention time
SILAC: stable isotope labeling by amino acids in cell culture
S/N: signal-to-noise ratio
SpC: spectral counting
TN: triple-negative
XIC: extracted ion chromatography.
