# Supplementary material for: Covariation of Peptide Abundances Accurately Reflects Protein Concentration Differences
Source: Mol Cell Proteomics. 2017 Mar 16;16(5):936–48. doi: 10.1074/mcp.O117.067728 (PMC5417831; doi:10.1074/mcp.O117.067728)
Supplement: Supplemental Data [file supp_16_5_936__index.html]

Covariation of Peptide Abundances Accurately Reflects Protein Concentration Differences — Covariation of Peptide Abundances Accurately Reflects Protein Concentration Differences — Protein Quantification Based on Covariation of Peptides — Supplemental Data 

# Covariation of Peptide Abundances Accurately Reflects Protein Concentration Differences

## Supplemental Data

- Supplementary Information (.docx, 1.2 MB) - Supplementary Discussion, Supplementary Figure S1-S6 and Supplementary Table S1
- Supplementary Table S2 (.xlsx, 6.4 MB) - Protein Quantification in 20 Mixtures set
- Supplementary Table S3 (.xlsx, 2.3 MB) - Diffacto estimated Protein Concentrations Breast Cancer CPTAC
- Supplementary Table S4 (.xlsx, 825 KB) - Diffacto estimated Protein Concentrations Breast Cancer MPIB
- Supplementary Table S5 (.xlsx, 33.9 MB) - Peptide Abundances in 20 Mixtures set
- Supplementary Table S6 (.xlsx, 3.7 MB) - De novo identified proteins' concentrations in iPRG2015 set
