# Supplementary material for: Covariation of Peptide Abundances Accurately Reflects Protein Concentration Differences
Source: Mol Cell Proteomics. 2017 Mar 16;16(5):936–48. doi: 10.1074/mcp.O117.067728 (PMC5417831; doi:10.1074/mcp.O117.067728)
Supplement: Supplemental Data [file 10.1074_O117.067728_mcp.O117.067728-1.docx]

**Supplementary Information to**

**“Covariation of Peptide Abundances Accurately Reflects Protein Concentration Differences”**

*Bo Zhang^a^, Mohammad Pirmoradian^a,b^, Roman Zubarev^a,*^ and Lukas Käll^c,*^*

^a^ Department of Medical Biochemistry and Biophysics, Karolinska Institutet, Scheeles väg 2, SE-17177 Solna, Sweden.

^b^ Department of Laboratory Medicine, Karolinska University Hospital Huddinge, SE-14186 Huddinge, Sweden.

^c^ Science for Life Laboratory, School of Biotechnology, Royal Institute of Technology−KTH, SE-17165 Solna, Sweden.

* To whom correspondence should be addressed:

Lukas Käll ([lukas.kall@scilifelab.se](mailto:lukas.kall@scilifelab.se)) or Roman Zubarev ([roman.zubarev@ki.se](mailto:roman.zubarev@ki.se))

Table of Contents

[Supplementary Discussions 3](#_Toc476493200)

[1. Discussion on quantification-centered proteomics 3](#_Toc476493201)

[2. Discussion on tests of significance 3](#_Toc476493202)

[Supplementary Notes 4](#_Toc476493203)

[Supplementary Note 1 | Design of label-free quantification experiments 4](#_Toc476493204)

[Supplementary Note 2 | False quantification rate 7](#_Toc476493205)

[Supplementary Note 3 | Up-regulated proteins associated with missense TP53 mutations in breast cancer 12](#_Toc476493206)

[Supplementary Note 4 | Proteomic signature of muscle-invasive bladder cancer 15](#_Toc476493207)

[Supplementary Table Titles 17](#_Toc476493208)

[Supplementary References 18](#_Toc476493209)

# Supplementary Discussions

## Discussion on quantification-centered proteomics

In recently years, modern LC-MS/MS instruments have brought unprecedented accuracy, precision, and throughput into comparative shotgun proteomics studies. However, traditional approaches to the analysis of data from label-free data-dependent acquisition (DDA) only use the small fraction of the data that is reliably identified by fragment spectra across all samples. This might lead to the incorrect conclusion that, for such types of experiments, the increased sample size is a hindering rather than an enabling factor. However, once that quantifications are brought to the center of proteomics data analysis, procedures for quantitative shotgun proteomics are in fact reproducible^1^. In the current study, we demonstrated that despite not finding fragment spectra by DDA to support each peptide across multiple conditions, we still obtained accurate relative protein quantifications. An alternative choice of performing quantitative measurements of peptides is data-independent acquisition (DIA), which has shown both high specificity and reproducibility^2^ and gained an increasing popularity. However, since the principle of summarizing protein abundances is the same in both DDA and DIA, we believe that the Diffacto approach should be applicable to either type of data.

1. Discussion on tests of significance

A common practice in comparative proteomics is to test differentially expressed proteins based on the intermediate quantities of protein abundances summarized in each experiment individually. Diffacto instead estimates protein abundances for the whole groups of samples by directly integrating peptide signals from multiple experiments. This is an approach that was inspired by the recently published PECA approach^3^, which converts a protein-level test into multiple peptide-level tests then integrate the statistics under the null hypotheses that the proteins have the same concentrations across the experimental conditions. Without any further corrections, this procedure becomes very sensitive, but unfortunately it inflates the resulting confidence estimates. We hence had to use a Monte Carlo-based permutation test to estimate the actual false discovery rates among our identifications.

When correcting *p* value for multiple testing one frequently end up with very short lists of significant differences in proteomics studies on small sample sizes. The increased sensitivity in Diffacto from integration of peptide-level statistics and exclusion of non-informative (unreliable) proteins makes the procedure less conservative.

# Supplementary Notes

## **Supplementary Note 1 |** Design of label-free quantification experiments

It is frequently claimed that data-dependent acquisition (DDA) of MS/MS is of low reproducibility as the technique is prone to not identify peptides across all samples, resulting in missing quantitative values. However, we have previously demonstrated^1^ that missing value is not an intrinsic problem with the DDA approach, as long as one make better use of precursor abundances. In the current study, we intentionally reduced the redundancy of MS/MS spectra by splitting the precursor m/z range into three segments that are almost non-overlapping for DDA in the three LC-MS/MS measurements of each sample^4^. It turned out that, even around two-thirds of the peptide identifications in each run were given by transferring from other runs based on LC-MS alignment and chromatographic feature matching, the proportion of missing values of peptide quantification was reasonably small (12.1%). Most of remaining missing values were the results of the detection limit of the instrument and the in-fact low peptide concentrations in the samples.

The concentration differences among the 20 mixtures were sufficiently large for the differential analysis and the estimation of linear dynamic range (Table S1). Showing in the Figure S1, within the linear dynamic range, most constituent peptides of BSA, especially the abundant ones, were precisely and accurately quantified. The good linearity of peptides’ responses to the highest concentration of BSA demonstrated the high capacity of our LC-MS/MS system, while the non-linear responses to low BSA concentrations made it possible to determine the lower limit of quantification (LOQ), which resulted in a linear dynamic range of maximum of 30x concentration difference (Figure S1). Such a limit may affect the accuracy of quantification for the mixtures with the lowest concentrations of yeast (mixture-1) or human (mixture-20) peptides.

**Table S1 |** Protein concentrations and MS/MS experimental settings for LFQ of 20 mixtures.

* Precursor m/z ranges: 375-481 Th (Low), 479-601 Th (Mid) and 599-1400 Th (High).

¶ Sample-11 from the first batch of experiments was used as the internal standard.

| **Mixture** | **Peptide amount (ng)** | | | | **MS/MS Precursor m/z range^*^** | **Experiment ID** | **Batch** |
| --- | --- | --- | --- | --- | --- | --- | --- |
|  | **Human** | **BSA** | **Yeast** | **Sum** |  |  |  |
| **1** | 1000 | 0.02 | 0.98 | 1001 | Low | 20160112_P1_SEG_LOW | 1 |
|  | 1000 | 0.02 | 0.98 | 1001 | Mid | 20160112_P1_SEG_MID | 1 |
|  | 1000 | 0.02 | 0.98 | 1001 | High | 20160112_P1_SEG_HIGH | 1 |
| **2** | 900 | 0.2 | 100.8 | 1001 | Low | 20160112_P2_SEG_LOW | 1 |
|  | 900 | 0.2 | 100.8 | 1001 | Mid | 20160112_P2_SEG_MID | 1 |
|  | 900 | 0.2 | 100.8 | 1001 | High | 20160112_P2_SEG_HIGH | 1 |
| **3** | 850 | 0.4 | 150.6 | 1001 | Low | 20160112_P3_SEG_LOW | 1 |
|  | 850 | 0.4 | 150.6 | 1001 | Mid | 20160112_P3_SEG_MID | 1 |
|  | 850 | 0.4 | 150.6 | 1001 | High | 20160112_P3_SEG_HIGH | 1 |
| **4** | 800 | 0.6 | 200.4 | 1001 | Low | 20160112_P4_SEG_LOW | 1 |
|  | 800 | 0.6 | 200.4 | 1001 | Mid | 20160112_P4_SEG_MID | 1 |
|  | 800 | 0.6 | 200.4 | 1001 | High | 20160112_P4_SEG_HIGH | 1 |
| **5** | 750 | 1 | 250 | 1001 | Mid | 20160112_P5_SEG_MID | 1 |
|  | 750 | 1 | 250 | 1001 | High | 20160112_P5_SEG_HIGH | 1 |
|  | 750 | 1 | 250 | 1001 | Low | 20160112_P5_SEG_LOW_160121063813 | 2 |
| **6** | 700 | 1.4 | 299.6 | 1001 | Low | 20160112_P6_SEG_LOW | 1 |
|  | 700 | 1.4 | 299.6 | 1001 | Mid | 20160112_P6_SEG_MID | 1 |
|  | 700 | 1.4 | 299.6 | 1001 | High | 20160112_P6_SEG_HIGH | 1 |
| **7** | 650 | 1.8 | 349.2 | 1001 | Low | 20160112_P7_SEG_LOW | 1 |
|  | 650 | 1.8 | 349.2 | 1001 | Mid | 20160112_P7_SEG_MID | 1 |
|  | 650 | 1.8 | 349.2 | 1001 | High | 20160112_P7_SEG_HIGH | 1 |
| **8** | 600 | 2.6 | 398.4 | 1001 | Low | 20160112_P8_SEG_LOW | 1 |
|  | 600 | 2.6 | 398.4 | 1001 | High | 20160112_P8_SEG_HIGH | 1 |
|  | 600 | 2.6 | 398.4 | 1001 | Mid | 20160112_P8_SEG_MID_160121160232 | 2 |
| **9** | 550 | 3.8 | 447.2 | 1001 | Mid | 20160112_P9_SEG_MID | 1 |
|  | 550 | 3.8 | 447.2 | 1001 | High | 20160112_P9_SEG_HIGH | 1 |
|  | 550 | 3.8 | 447.2 | 1001 | Low | 20160112_P9_SEG_LOW_160121012404 | 2 |
| **10** | 500 | 5.4 | 495.6 | 1001 | Mid | 20160112_P10_SEG_MID | 1 |
|  | 500 | 5.4 | 495.6 | 1001 | High | 20160112_P10_SEG_HIGH | 1 |
|  | 500 | 5.4 | 495.6 | 1001 | Low | 20160112_P10_SEG_LOW_160120200540 | 2 |
| **11** | 450 | 7.6 | 543.6 | 1001 | Low | 20160112_P11_SEG_LOW_160203031257 | 3 |
|  | 450 | 7.6 | 543.6 | 1001 | Mid | 20160112_P11_SEG_MID_160203050927 | 3 |
|  | 450 | 7.6 | 543.6 | 1001 | High | 20160112_P11_SEG_HIGH_160203070611 | 3 |
| **12** | 400 | 10.6 | 590.4 | 1001 | Low | 20160112_P12_SEG_LOW | 1 |
|  | 400 | 10.6 | 590.4 | 1001 | Mid | 20160112_P12_SEG_MID | 1 |
|  | 400 | 10.6 | 590.4 | 1001 | High | 20160112_P12_SEG_HIGH | 1 |
| **13** | 350 | 15 | 636 | 1001 | Low | 20160112_P13_SEG_LOW | 1 |
|  | 350 | 15 | 636 | 1001 | Mid | 20160112_P13_SEG_MID | 1 |
|  | 350 | 15 | 636 | 1001 | High | 20160112_P13_SEG_HIGH | 1 |
| **14** | 300 | 21.2 | 679.8 | 1001 | Mid | 20160112_P14_SEG_MID | 1 |
|  | 300 | 21.2 | 679.8 | 1001 | High | 20160112_P14_SEG_HIGH | 1 |
|  | 300 | 21.2 | 679.8 | 1001 | Low | 20160112_P14_SEG_LOW_160120174525 | 2 |
| **15** | 250 | 30 | 721 | 1001 | Low | 20160112_P15_SEG_LOW | 1 |
|  | 250 | 30 | 721 | 1001 | Mid | 20160112_P15_SEG_MID | 1 |
|  | 250 | 30 | 721 | 1001 | High | 20160112_P15_SEG_HIGH_160120220930 | 2 |
| **16** | 200 | 42.4 | 758.6 | 1001 | Low | 20160112_P16_SEG_LOW | 1 |
|  | 200 | 42.4 | 758.6 | 1001 | Mid | 20160112_P16_SEG_MID | 1 |
|  | 200 | 42.4 | 758.6 | 1001 | High | 20160112_P16_SEG_HIGH_160121181003 | 2 |
| **17** | 150 | 60 | 791 | 1001 | Low | 20160112_P17_SEG_LOW | 1 |
|  | 150 | 60 | 791 | 1001 | Mid | 20160112_P17_SEG_MID | 1 |
|  | 150 | 60 | 791 | 1001 | High | 20160112_P17_SEG_HIGH | 1 |
| **18** | 100 | 84.8 | 816.2 | 1001 | Low | 20160112_P18_SEG_LOW | 1 |
|  | 100 | 84.8 | 816.2 | 1001 | Mid | 20160112_P18_SEG_MID | 1 |
|  | 100 | 84.8 | 816.2 | 1001 | High | 20160112_P18_SEG_HIGH | 1 |
| **19** | 50 | 120 | 831 | 1001 | Low | 20160112_P19_SEG_LOW | 1 |
|  | 50 | 120 | 831 | 1001 | High | 20160112_P19_SEG_HIGH | 1 |
|  | 50 | 120 | 831 | 1001 | Mid | 20160112_P19_SEG_MID_160121112852 | 2 |
| **20** | 1 | 0.1 | 999.9 | 1001 | Low | 20160112_P20_SEG_LOW | 1 |
|  | 1 | 0.1 | 999.9 | 1001 | Mid | 20160112_P20_SEG_MID | 1 |
|  | 1 | 0.1 | 999.9 | 1001 | High | 20160112_P20_SEG_HIGH_160121032454 | 2 |
| **Reference^¶^** | 350 | 7.6 | 636 | 993.6 | Low | 20160112_P11_SEG_LOW | 1 |
|  | 350 | 7.6 | 636 | 993.6 | Mid | 20160112_P11_SEG_MID | 1 |
|  | 350 | 7.6 | 636 | 993.6 | High | 20160112_P11_SEG_HIGH | 1 |

**
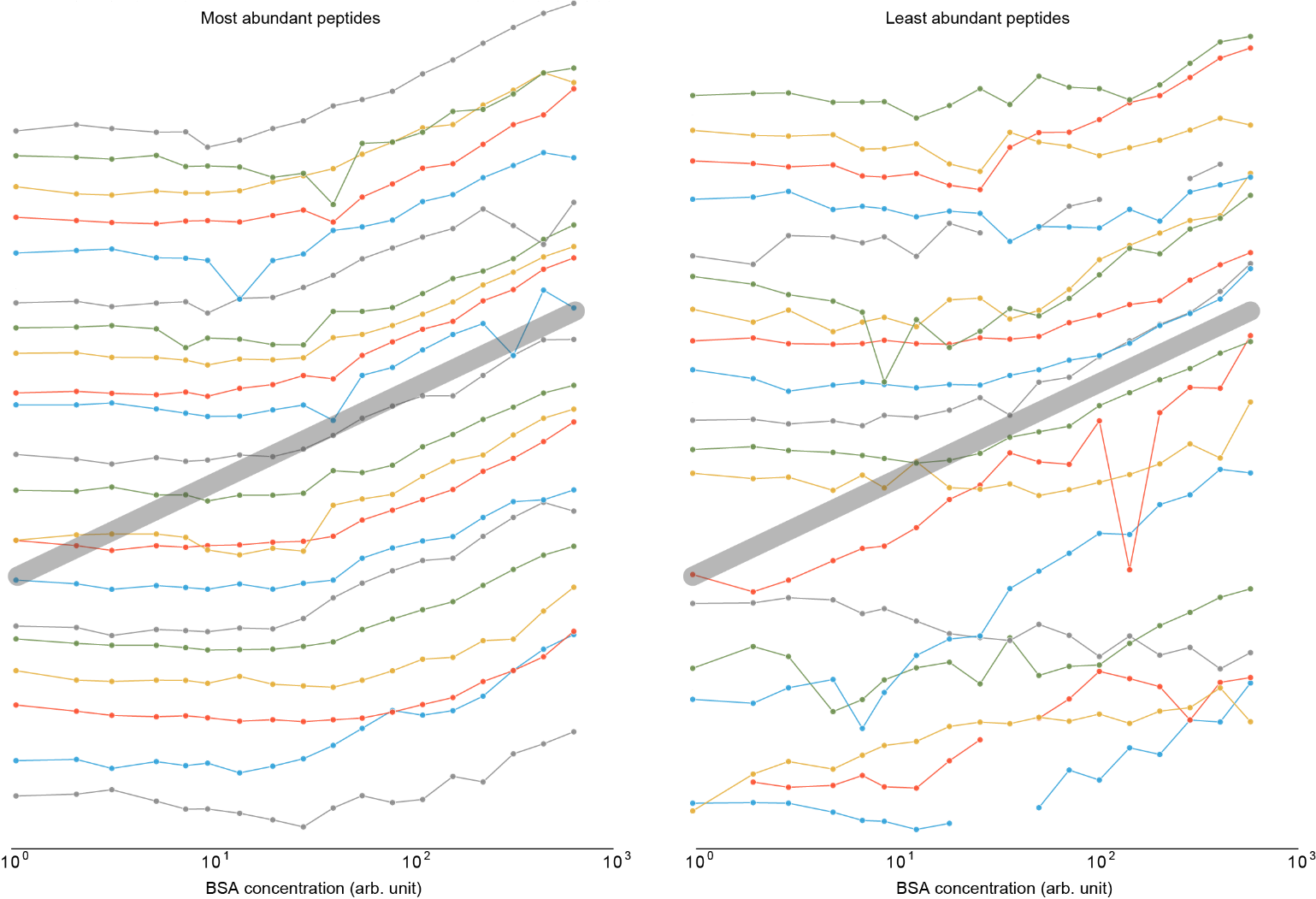
**

**Figure S1 |** Relative abundances changes of 40 BSA-derived peptides quantified in 18 mixture samples with BSA concentrations ranging from 1 to 600 arbitrary units (ng / 30 μL). Peptides were sorted by the median abundances with a constant vertical distance. Grey bands represent the expected linear trend of peptide abundances in response to the protein concentration changes. Peptides with higher abundances (left panel) showed a more linear response than that of lower abundant peptides (right panel). The lower limit of quantification was observed in half of the samples with low BSA concentrations. Therefore, the linear dynamic range of label-free quantification was estimated at a maximum of 30x difference in this experiment.

## **Supplementary Note 2 |** False quantification rate

Generally, proteins with a high concentration produce a larger number of peptides with better chromatographic features and higher quality fragment spectra than proteins with a low concentration. Consequently, proteins with a high concentration have a higher chance to be associated with correctly matched peptide-spectrum matches. Also, the impact of incorrect assignments of peptide identities will be proportionally reduced by the total number of constituent peptides (Figure S2a). However, it should be noted that there might be other explanations for non-correlating peptides in complex proteomic samples than false quantifications. For example we might observe differentially expressed protein isoforms^5^ , in which case the linear model of protein quantification will not be a viable model.

From the covariation structure, Diffacto’s factor analysis provides a quality threshold for detecting peptides that are not correlating with the underlying factor. Estimated from the 20 mixture LFQ dataset, around 10% of peptides were categorized as non-correlating, which introduced large variances in the protein-level aggregation as showed in Figure S3. False quantifications results estimated from anti-correlation between the summarized protein abundances and the actual protein concentration (see Figure 1) also showed significant dependency to the number of peptides. The S/N filtering showed its effectiveness of removing false quantifications from non-informative results.

a


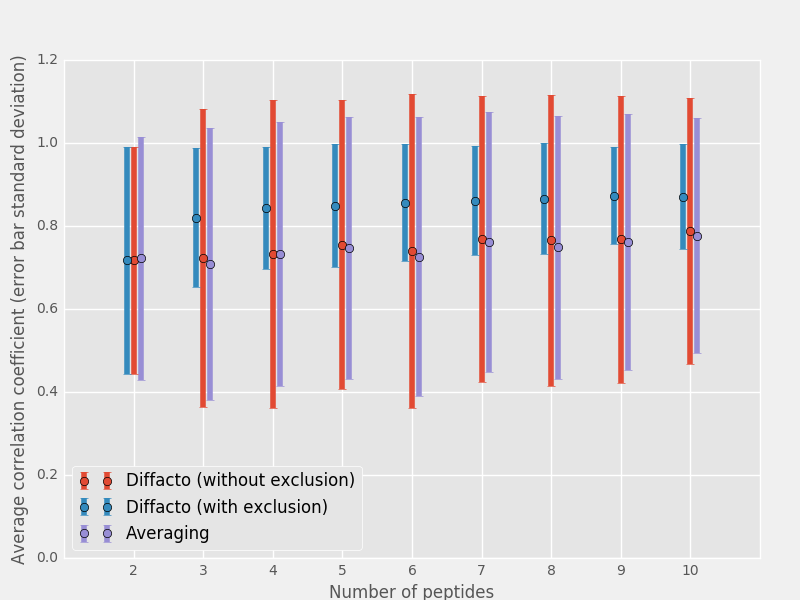


b

c

**Figure S2 |** **a)** Auto exclusion of non-correlating peptides by factor analysis increases overall correlation between peptides abundances and the summarized quantification result. (Error-bar: standard deviation). **b)** Comparison of false quantification rates (before S/N filtering). **c)** Comparison of false quantification rates (after S/N > 0.01 filtering).

**
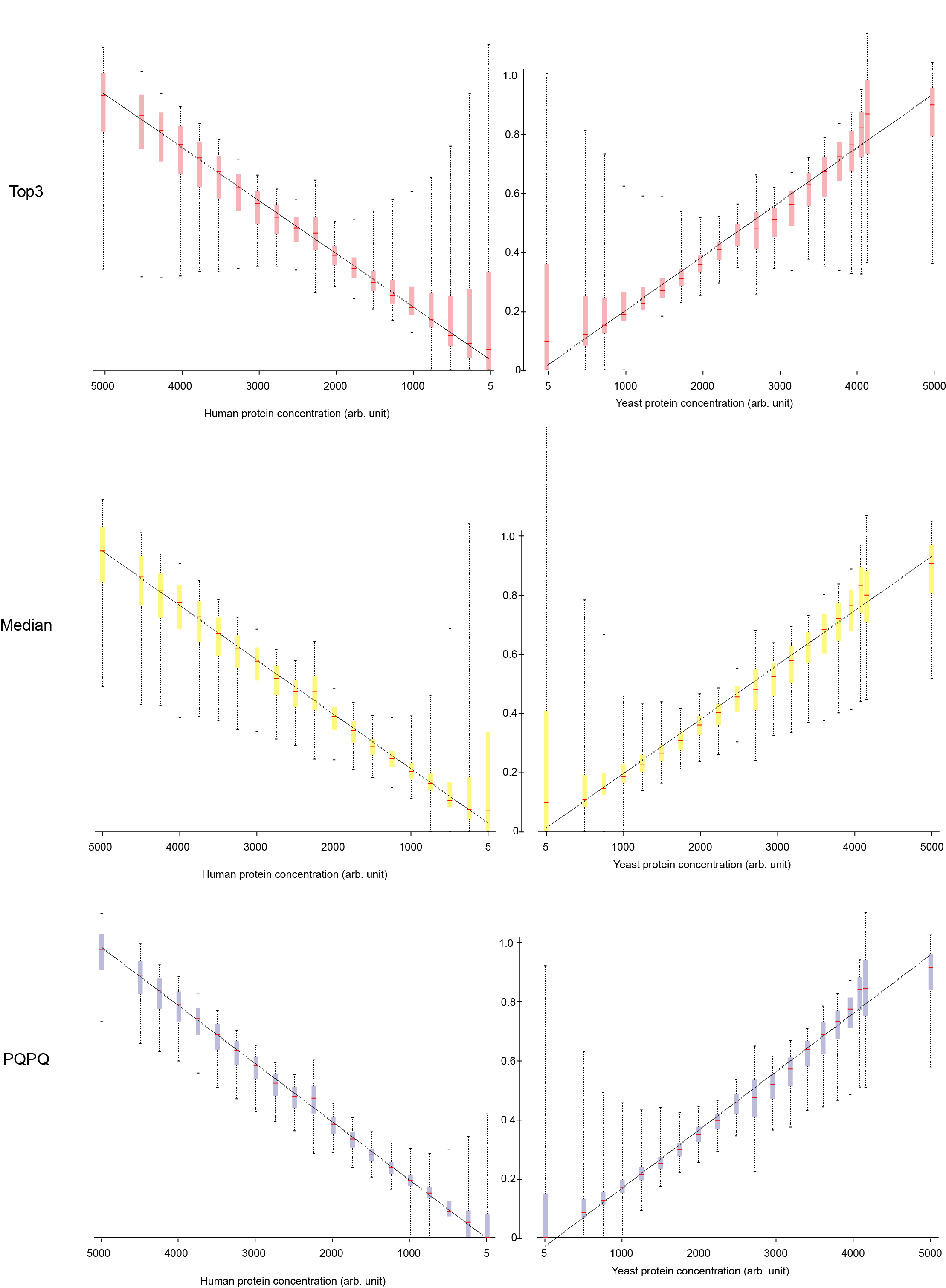
**

**Figure S3 |** Relative protein concentrations summarized by experiment-wise averaging of Top-3 (red) peptide abundances; Median (yellow) peptide abundances; or averaging of PQPQ filtered correlating peptides. In comparison, the Diffacto summarized protein concentrations error margins (Figure 1c) are visibly lower than the error margins of any of these three methods. Arbitrary unit (ng / 30 μL).

## **Supplementary Note 3 |** Up-regulated proteins associated with missense TP53 mutations in breast cancer

For each sample, the CPTAC data provided the mutation status of the TP53 gene. This enabled us to investigate the impact of TP53 mutations on the proteome, to shed light on why the progression of breast cancers to such a high degree hinge on missense mutation of that particular gene. Hence, we re-labeled the samples based on their TP53 status as missense mutation, frameshift/nonsense mutation, and NA (presumed to be normal), and performed another three-group Diffacto analysis.

For the missense mutation of TP53, the most specific regulation is the over-expression of the point-mutated TP53 itself, which is in accordance with Mertins et al.^6^. However, as ERPR+ samples were dominant in the group with normal TP53, proteome signatures associated to the ERPR+ subtype may also be associated with normal TP53. It reflected the fact that the ERPR+ (or specifically the Luminal A subtype) breast cancer might be least affected by TP53, which makes the ERPR+ samples confounders in detecting TP53-related proteome changes. Consequently, many proteins associated to TP53 mutations failed to pass our self-imposed 5% FDR threshold as estimated by a Monte Carlo tests, but were still possible to detect by the sensitive peptide-level approach (PECA^3^). Indeed, we observed strong evidences from the peptide-level analysis that most of the TP53-associated proteins, including S100A8, S100A9, S100A12, MMP8, MMP9, RETN, PADI4, BPI, CAMP, ELANE, DEFA1, DEFA3, MPO, AZU1, PRTN3, EPX, LTF, RNASE3, TCN1, etc., were up-regulated exclusively in the ER-negative subtypes (Supplementary Figure S4). On top of the list, we also confirmed a known biomarker associated with progression of ER-negative breast cancer: Lipocalin 2 (LCN2, also known as oncogene 24p3)^7^. Interestingly, many of these TP53-specific markers were classified in the original study as “basal-enriched” by the analysis of co-expression (see Extended Data Figure 8 of Mertins et al.). Based on the results from Diffacto, we could suggest an alternative theory that a point-mutated TP53 might regulate the co-expression of these proteins, and potentially cause the basal-like cancer phenotype.


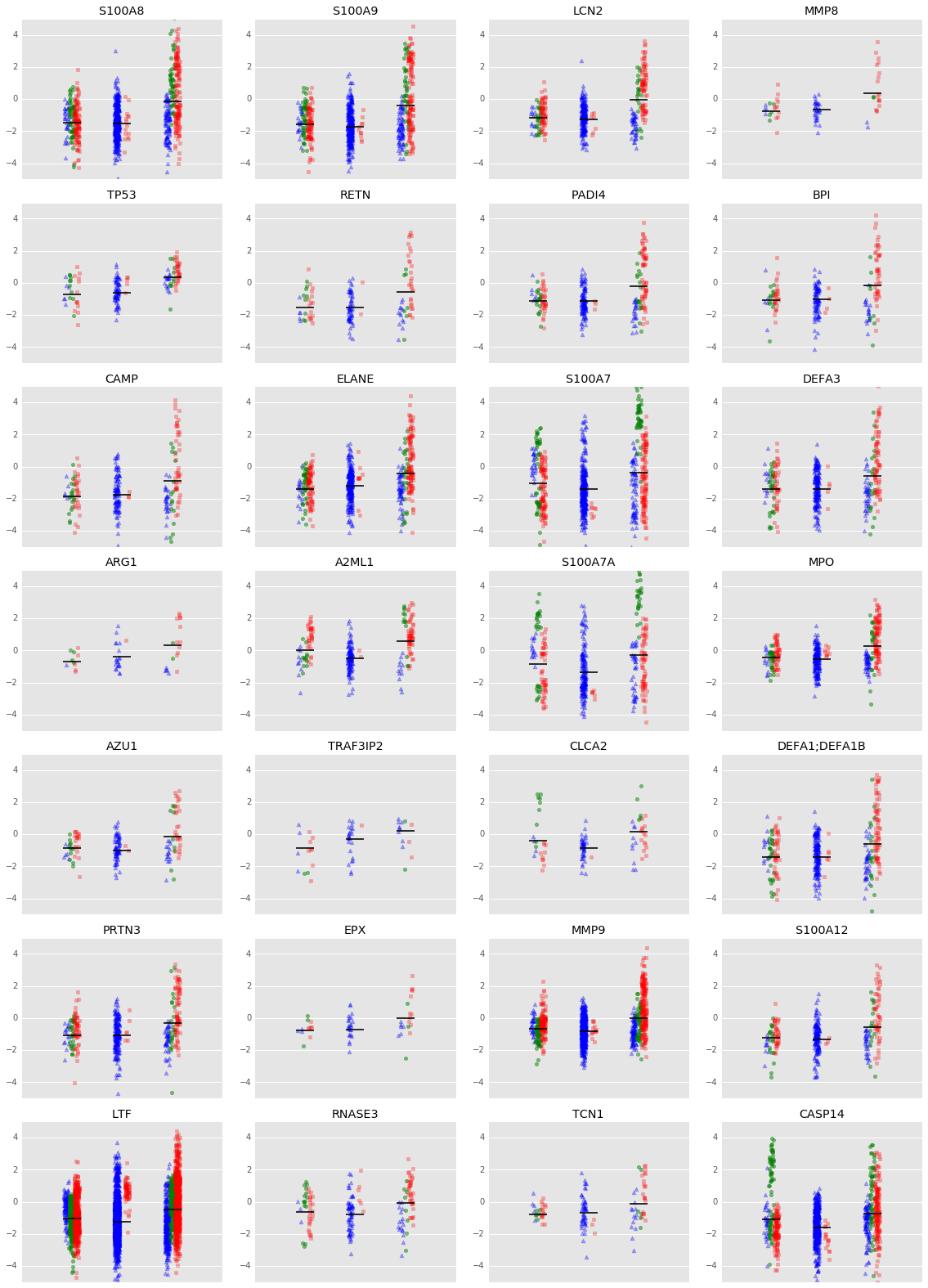


**Figure S4 |** The most up-regulated proteins related to TP53 point mutation. In each sub-figure, relative peptide abundances (log ratios) were grouped by TP53 mutation status (left: frameshift/nonsense mutation, middle: normal, right: missense mutation), and color coded by cancer subtypes (ERPR+: blue triangles, HER2+: green dots, TN: red squares). Normal TP53 (middle) group does not contain HER2+ (green) sample. Y-axis: relative abundances in log2-scale. Black bars: weighted averages of TP53 groups.

**
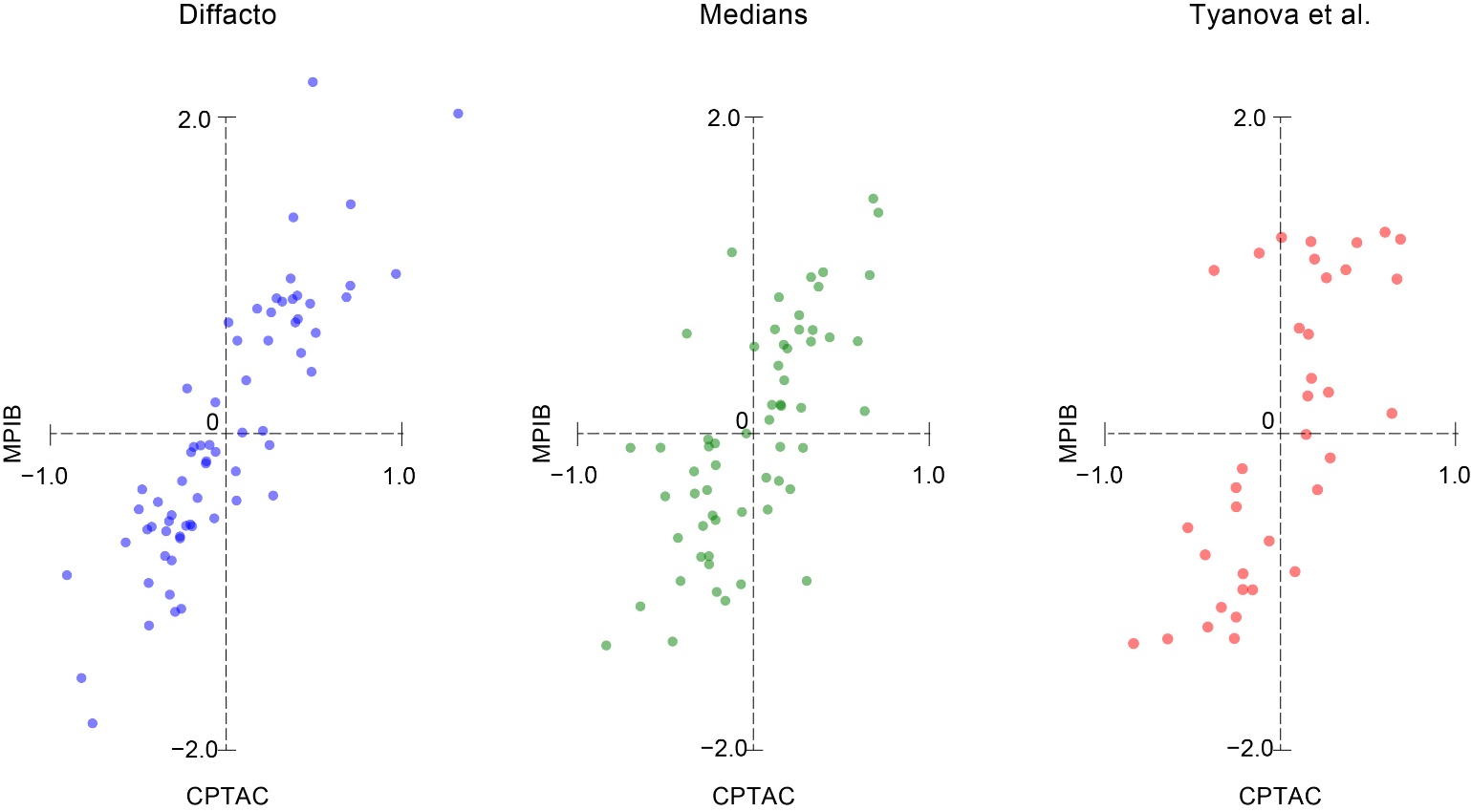
**

**Figure S5** | Consistency of relative protein quantification (log_2_-fold-changes) between CPTAC^6^ and MPIB^8^. Left panel: comparison of relative protein abundances (weighted geometric means) of 22 proteins (exclusively detected by Diffacto) in three subtypes of breast cancer; Middle panel: comparison of relative protein abundances (median protein ratios) of 20 proteins (detected by the traditional median-based approach or the original study^8^) in the three subtypes; Right panel: comparison of relative protein abundances (median protein ratios) of 12 proteins (exclusively reported in the original study) in the three subtypes. A stronger linear correlation (r = 0.88) was given by the Diffacto results, compared to that of the other two approaches (r = 0.70 in both cases). Protein ratios estimated from MPIB data were generally larger than the ratios from CPTAC data, likely due to the ratio distortion effect in iTRAQ caused by the co-isolation of precursors^9, 10^.

## **Supplementary Note 4 |** Proteomic signature of muscle-invasive bladder cancer

In principle, larger sample size and higher sequence coverage in terms of more peptides per protein should give a clearer measure of covariation. However, to demonstrate that Diffacto also can improve protein quantification in clinical studies with fewer samples and modest sequence coverage, we applied Diffacto to a clinical dataset of bladder cancer previously described by Latosinska et al^11^. These data were acquired by different quantification techniques (LFQ and 8-plex iTRAQ labeling with and without sample fractionation), pairwise comparing the muscle-invasive (stage T2+) and non-muscle invasive (stage Ta) cancer subtypes. The peptide abundances were re-normalized by the median value of non-missing measurements in each experiment.

Due to the small sample size, Monte Carlo random permutation tests were not effective for estimating FDR. Hence, we only applied the PECA approach^3^ that integrates peptide-level statistics for detecting differentially expressed proteins, and obtained in total 178 significant proteins (p < 0.05); 35 of which overlapped between LFQ and iTRAQ results (Figure S6a). Compared to the original numbers report by Latosinska et al., the peptide-level integration was more sensitive. The linear correlation between LFQ and iTRAQ for the 35 protein ratios was 0.93 (Pearson’s r) indicated high reproducibility of shotgun proteomics, regardless of the technical choices for quantiﬁcation (Figure S6a). LFQ tended to give larger ratios than iTRAQ, likely due to the effect of ratio distortion in iTRAQ^9, 10^, or the imputation of missing values in LFQ data (Online Methods).

We performed pathway enrichment analysis on the DAVID bioinformatics platform (v6.8)^12^, by submitting the list of the 178 proteins summarized by Diffacto as “gene list” and all quantified proteins as “background”. KEGG pathways with FDR < 0.05 (Benjamini) were considered as significantly enriched, which showed significant alterations of glutathione and carbon metabolism (Figure S6b). Based on such a partial picture of the proteome we could develop a theory that the invasive nature of the cancer is associated to the cellular responses to oxidative stress, which was characterized by the drastic down-regulation of glutathione S-transferases (which are known markers of bladder cancer development^13^) and up-regulation of PGD, IDH2, G6PD and PHGDH, indicating biochemical processes being promoted for serine biosynthesis, NADPH production and glutathione utilization^14^.


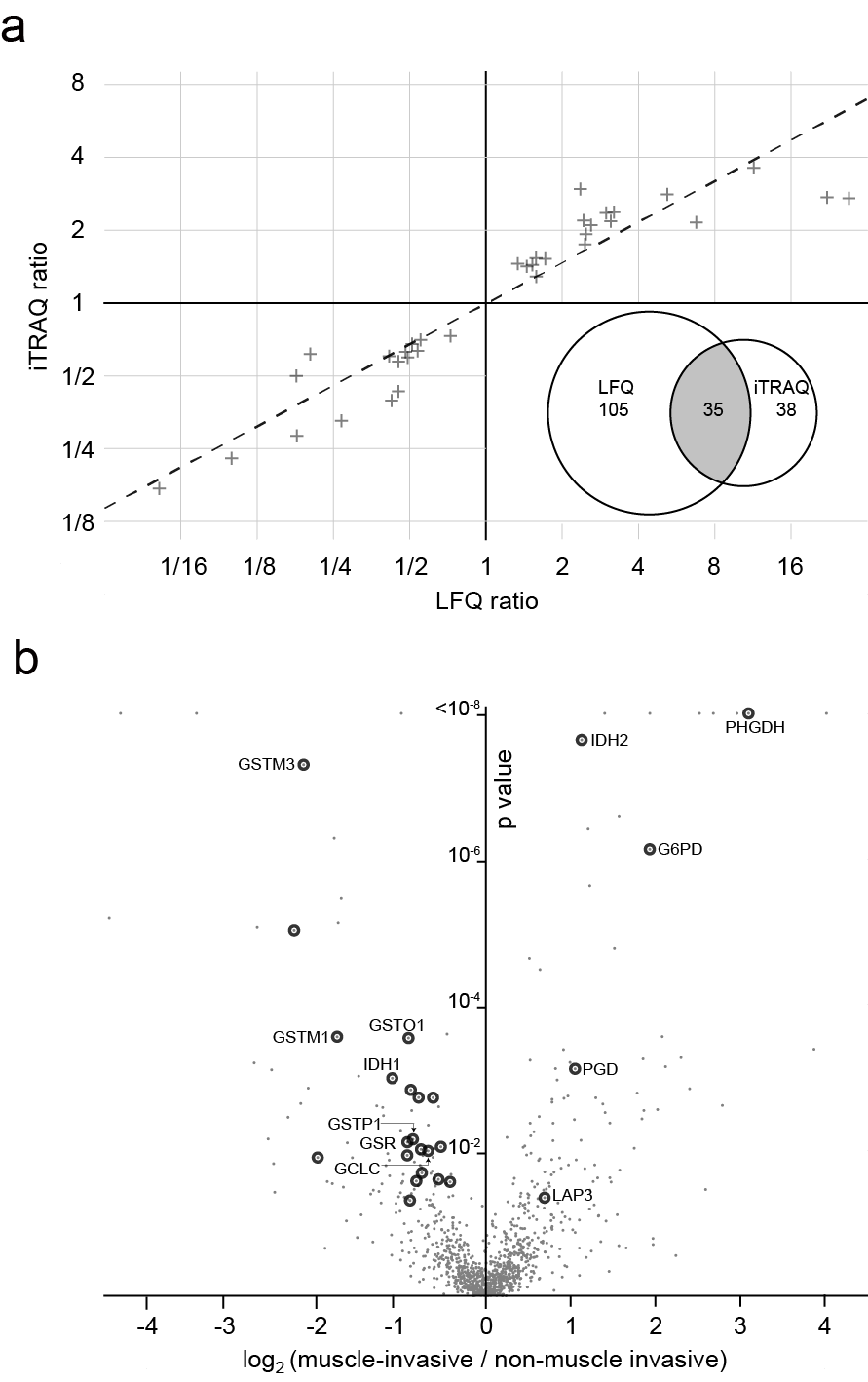


**Figure S6 | Proteome comparison between two bladder cancer subtypes. (a)** Comparison of abundance ratios from LFQ and iTRAQ data, using the 35 common proteins (Venn chart) summarized by Diffacto (p < 0.05). **(b)** Differentially expressed proteins participating in glutathione and carbon metabolism pathways (circles) were mostly down-regulated in muscle-invasive bladder cancer. Proteins related to antioxidant response are labeled in the volcano plot.

# Supplementary Table Titles

**Supplementary Table S2 |** Label-free protein quantification of the 20 mixtures

**Supplementary Table S3 |** Diffacto result for the CPTAC breast cancer dataset

**Supplementary Table S4 |** Diffacto result for the MPIB breast cancer dataset

**Supplementary Table S5 |** Label-free peptide quantification of the 20 mixtures

**Supplementary Table S6 |** Quantification for iPRG-2015 based on peptide *de novo* sequencing

# Supplementary References

1. Zhang, B., Käll, L. & Zubarev, R.A. DeMix-Q: Quantification-Centered Data Processing Workflow. *Mol Cell Proteomics* **15**, 1467-1478 (2016).

2. Navarro, P. et al. A multicenter study benchmarks software tools for label-free proteome quantification. *Nat Biotechnol* **34**, 1130-1136 (2016).

3. Suomi, T., Corthals, G.L., Nevalainen, O.S. & Elo, L.L. Using Peptide-Level Proteomics Data for Detecting Differentially Expressed Proteins. *J Proteome Res* **14**, 4564-4570 (2015).

4. Vincent, C.E. et al. Segmentation of precursor mass range using "tiling" approach increases peptide identifications for MS1-based label-free quantification. *Analytical chemistry* **85**, 2825-2832 (2013).

5. Lukasse, P.N.J. & America, A.H.P. Protein Inference Using Peptide Quantification Patterns. *Journal of Proteome Research* **13**, 3191-3199 (2014).

6. Mertins, P. et al. Proteogenomics connects somatic mutations to signalling in breast cancer. *Nature* **534**, 55-62 (2016).

7. Yang, J. et al. Lipocalin 2 promotes breast cancer progression. *Proc Natl Acad Sci U S A* **106**, 3913-3918 (2009).

8. Tyanova, S. et al. Proteomic maps of breast cancer subtypes. *Nature communications* **7**, 10259 (2016).

9. Ow, S.Y. et al. iTRAQ underestimation in simple and complex mixtures: "the good, the bad and the ugly". *J Proteome Res* **8**, 5347-5355 (2009).

10. Zhang, B., Pirmoradian, M., Chernobrovkin, A. & Zubarev, R.A. DeMix workflow for efficient identification of cofragmented peptides in high resolution data-dependent tandem mass spectrometry. *Mol Cell Proteomics* **13**, 3211-3223 (2014).

11. Latosinska, A. et al. Comparative Analysis of Label-Free and 8-Plex iTRAQ Approach for Quantitative Tissue Proteomic Analysis. *PLoS One* **10**, e0137048 (2015).

12. Huang, D.W., Sherman, B.T. & Lempicki, R.A. Systematic and integrative analysis of large gene lists using DAVID bioinformatics resources. *Nature Protocols* **4**, 44-57 (2008).

13. Schnakenberg, E., Breuer, R., Werdin, R., Dreikorn, K. & Schloot, W. Susceptibility genes: GSTM1 and GSTM3 as genetic risk factors in bladder cancer. *Cytogenetics and cell genetics* **91**, 234-238 (2000).

14. Gorrini, C., Harris, I.S. & Mak, T.W. Modulation of oxidative stress as an anticancer strategy. *Nat Rev Drug Discov* **12**, 931-947 (2013).
